# Dynamics of Cell-Fate Determination and Patterning in the Vascular Bundles of *Arabidopsis thaliana*

**DOI:** 10.1371/journal.pone.0063108

**Published:** 2013-05-27

**Authors:** Mariana Benítez, Jan Hejátko

**Affiliations:** 1 Functional Genomics and Proteomics of Plants, Central European Institute of Technology, Masaryk University, Brno, Czech Republic; 2 Departamento de Ecología de la Biodiversidad, Instituto de Ecología, Universidad Nacional Autónoma de México, México DF, Mexico; 3 Centro de Ciencias de la Complejidad C3, Universidad Nacional Autónoma de México, México DF, Mexico; Universidad Miguel Hernández de Elche, Spain

## Abstract

Plant vascular meristems are sets of pluripotent cells that enable radial growth by giving rise to vascular tissues and are therefore crucial to plant development. However, the overall dynamics of cellular determination and patterning in and around vascular meristems is still unexplored. We study this process in the shoot vascular tissue of *Arabidopsis thaliana*, which is organized in vascular bundles that contain three basic cell types (procambium, xylem and phloem). A set of molecules involved in this process has now been identified and partially characterized, but it is not yet clear how the regulatory interactions among them, in conjunction with cellular communication processes, give rise to the steady patterns that accompany cell-fate determination and arrangement within vascular bundles. We put forward a dynamic model factoring in the interactions between molecules (genes, peptides, mRNA and hormones) that have been reported to be central in this process, as well as the relevant communication mechanisms. When a few proposed interactions (unverified, but based on related data) are postulated, the model reproduces the hormonal and molecular patterns expected for the three regions within vascular bundles. In order to test the model, we simulated mutant and hormone-depleted systems and compared the results with experimentally reported phenotypes. The proposed model provides a formal framework integrating a set of growing experimental data and renders a dynamic account of how the collective action of hormones, genes, and other molecules may result in the specification of the three main cell types within shoot vascular bundles. It also offers a tool to test the necessity and sufficiency of particular interactions and conditions for vascular patterning and yields novel predictions that may be experimentally tested. Finally, this model provides a reference for further studies comparing the overall dynamics of tissue organization and formation by meristems in other plant organs and species.

## Introduction

During their embryonic and postembryonic development, plants continuously generate new tissues, organs and structures from pools of undifferentiated cells that are located in specialized structures called meristems. Thus, studying the formation and maintenance of the spatiotemporal patterns that underlie cell-fate determination and arrangement within and around the meristems is of special importance for understanding plant development. Indeed, experimental and theoretical approaches have begun to uncover the complex interactions among genes, hormones and other molecules involved in the formation and patterning of root and shoot apical meristems of *Arabidopsis thaliana* (Arabidopsis) [1]–[3]. Less is known, however, about the dynamics of cellular patterning in the vascular meristem, which gives rise to vascular tissues and underlies plant growth in the radial direction.

**Figure 1:**
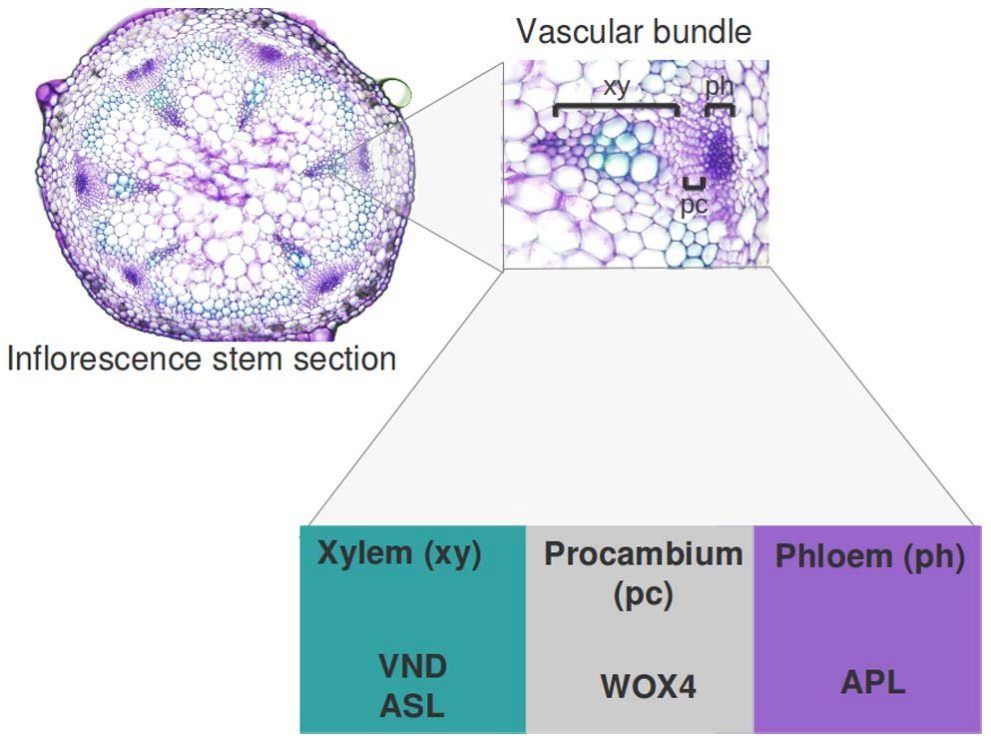
Schematic representation of the vascular bundle structure. The shoot vascular bundles of Arabidopsis are considered as an array of three compartments in the position of procambial (pc) and young xylem (xy) and phloem (ph) cells. Each cell type is characterized by, among other things, the presence of one or two proteins that we will consider cell-type markers: VND and ASL in the xylem, WOX4 in the procambium, and APL in the phloem. The microphotograph is showing the cross section of living (non-fixed) inflorescence stem of Arabidopsis stained with toluidine blue as described in [15].

Although the structures of Arabidopsis vascular tissues are different in the root than in the shoot, primary vascular tissues of both plant portions consist of meristematic procambial cells that produce xylem cells on one side and phloem cells on the other (Figure 1) [4]. Vascular bundles of the inflorescence stem are formed by the inflorescence apical meristem in a process known as primary growth. First, procambium differentiates to form phloem and, later, xylem [5]. Later in the development, the secondary meristem – cambium – differentiates in both vascular bundles and interfascicular regions, giving rise to the secondary phloem and xylem. In Arabidopsis, the secondary growth is limited to the very base of the inflorescence stem and is initiated in the phase of the first silique formation [6]. Here, we focus on the study of cellular determination and patterning of the three basic cell types during primary growth in the shoot vasculature (Figure 1). Specifically, we study the maintenance of procambial cells and determination of the young xylem and phloem cells situated next to them. Although a set of molecular elements involved in this process has been identified (Table 1 in File S1), it is not known how the regulatory interactions among these elements, in conjunction with cell-to-cell communication processes [7], render the steady patterns that characterize the three regions within vascular bundles. In order to explore this process, we put forward a data-based spatiotemporal model considering the interactions among genes, peptides, mRNA and hormones that have been reported to be central to this process (Tables 1 and 2 in File S1).

Among plant hormones, auxin and cytokinins (IAA and CKs, respectively) play an important role in the specification and patterning of shoot and root vascular cells (see reviews in [8]–[10]). In our work we further consider brassinosteroids (BRs), which also regulate vascular development [8], [11] but whose role in vascular cell-fate determination within bundles remains unclear.

IAA is necessary for the specification of vascular cell precursors and is found in procambial cells, although not exclusively. In the inflorescence stem of Arabidopsis, IAA reporters are observed in the procambial and differentiating xylem [11]. This pattern has also been observed in the root vasculature [12], [13]. In both cases, IAA signal appears to sustain procambial identity and promote xylem differentiation [9], [10], [13], probably via *MONOPTEROS* (*MP*) and other *AUXIN RESPONSE FACTORS* (*ARFs*) (Table 1 in File S1 and references therein).

**Figure 2:**
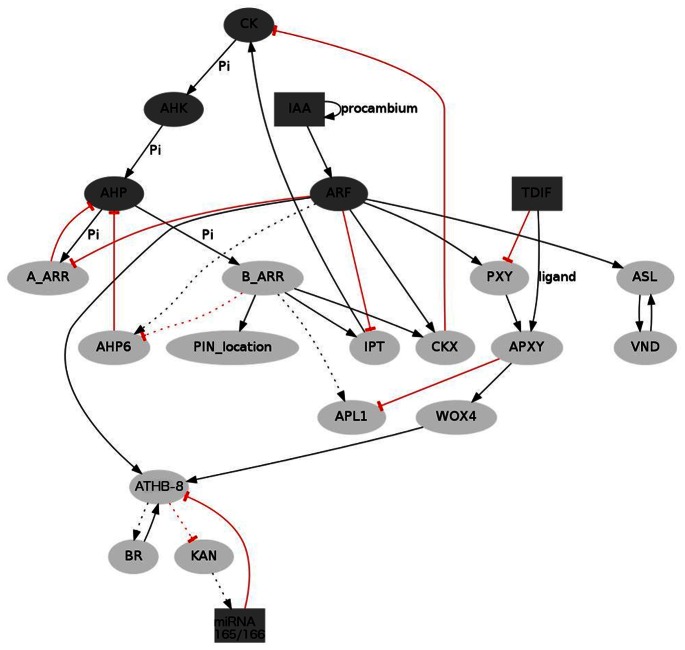
Summary of the experimental data used to specify the dynamic model. Nodes correspond to different types of molecular regulatory interactors; arrows represent activation or positive regulation. Red, flat-end lines represent negative regulation. Nodes can take on only two values (0 or 1), except for the dark gray ones which can take on three (0, 1, 2). Mobile elements are represented in squares, and dotted lines indicate hypothetical interactions.

CKs are also present in vascular procambial cells and positively regulate their proliferation [14]–[16] while negatively regulating protoxylem specification [17]. As currently understood, CK signaling is mediated by a multistep phosphorelay (MSP) system. This system includes hybrid sensor histidine kinases (*AHKs*), histidine phosphotransfer proteins (*AHPs*), and nuclear response regulators (*ARRs*) (for a recent review see [18]). ARRs are subdivided into several groups, the most important of these being A-type (*A-ARRs*) and B-type (*B-ARRs*). In turn, ARRs regulate the expression of other genes, including isopentenyl transferases (*IPT)* and cytokinin dehydrogenases (*CKX)*, which regulate CK biosynthesis and degradation, respectively (Figure 2, Table 1 in File S1, and references therein).

It was recently shown that in the root vasculature of Arabidopsis CKs and IAA mutually determine their spatial distribution. IAA upregulates *AHP6*, a negative regulator of the CK signaling system, while CKs upregulate the auxin efflux transporter PIN7 [13]. Although this mechanism has not been reported for the shoot, the known regulatory interactions and spatial patterns of these elements in the shoot of Arabidopsis do not exclude it (Tables 1 and 2 in File S1).

In addition to the growth regulators mentioned above, there exists a set of genes and molecules involved in cell-fate determination and patterning within vascular bundles of Arabidopsis (Figure 2 and Table 1 in File S1). This set includes the HD-ZIP III gene *ATHB-8*, which is expressed in preprocambial and procambial cells [19], [20], and *KANADI* (*KAN*), which has been shown to downregulate HD-ZIP III genes in other contexts and has been suggested to downregulate *ATHB-8* via miRNA165/6 (MIR) [10]. The xylem-specific genes *VASCULAR-RELATED NAC-DOMAIN* (*VND*) and *ASYMMETRIC LEAVES2-LIKE19* (*ASL*) have also been included [10], [21], [22], as well as the phloem-specific *ALTERED PHLOEM DEVELOPMENT* (*APL*) gene [23]. Finally, we considered the procambium-specific gene *WUSCHEL-RELATED HOMEOBOX 4* (*WOX4*) [24] and the peptide TRACHEARY ELEMENT DIFFERENTIATION INHIBITORY FACTOR (TDIF) that binds the receptor-like kinase PHLOEM INTERCALATED WITH XYLEM (PXY) and activates it (the activated form is denoted here as *APXY*). Importantly, TDIF and miRNA165/6 are able to diffuse between neighboring cells [7], [25]–[27].

Since the molecules that have been reported as necessary for the patterning of vascular bundles regulate one another in a complex (i.e. highly nonlinear) manner, at both the intra- and intercellular scales, mathematical and computational models are expected to be helpful in understanding such patterning processes, as well as for integrating vast sets of detailed experimental data and providing novel predictions. Among those mathematical models put forward in developmental biology are those of molecular regulatory networks (MRN) (e.g. [28]). These are integrative tools that allow assessing the collective action of several interacting molecules and enable simulations of modified and hypothetical networks. MRN models consider genes, proteins or other molecules as nodes, and they regard regulatory interactions as directed edges (positive or negative). We thus developed a dynamic spatiotemporal model that allowed us to i) generate a formal framework that serves as a basis for integrating the currently available experimental data, as well as the continuously emerging data; ii) postulate a dynamic account of how the collective action of hormones, genes and other molecules results in specification of the three main regions and cell types within vascular bundles; iii) put forward novel predictions that can be experimentally tested; and iv) establish a reference model that can be used for further studies comparing the dynamics of patterning and meristem maintenance.

Our results render a dynamic mechanism that could account for vascular bundle patterning. It also suggests that in the shoot vasculature, CKs and IAA exclude one another in a way similar to that proposed for the root vasculature [13]. Additionally, the model points to some testable interactions that could occur either directly or indirectly and that may feed back to experimental work: that CKs upregulate APL, that KAN and ATHB-8 downregulate each other via MIR165/6, that ATHB-8 self-upregulates, and that AHP6 is regulated in the shoot as it is regulated in the root (positively by IAA and negatively by CKs). Finally, our model enables further comparative analyses with patterning systems in other meristematic regions of Arabidopsis and in other plant systems (e.g. *Zinnia elegans* [Zinnia]), providing a basis for the comparison of whole dynamic developmental modules [29].

## Methods

The proposed model considers data that we identified and evaluated through an extensive search (up to January 2012). It takes into account molecular interactions, hormonal and expression patterns, and cell-to-cell communication processes that have been reported to affect vascular patterning in the bundles of Arabidopsis. The model components and interactions are graphically presented in Figure 2, and the evidence supporting the model is summarized in Tables 1 and 2 in File S1.

In the network model, nodes stand for molecular elements regulating one another’s activities. Most of the nodes can take only 1 or 0 values (light gray nodes in Figure 2), corresponding to “present” or “not present,” respectively. Since the formation of gradients of hormones and diffusible elements may have important consequences in pattern formation, mobile elements TDIF and MIR, as well as members of the CK and IAA signaling systems, can take 0, 1 or 2 values (dark gray nodes in Figure 2). The level of expression for a given node is represented by a discrete variable *g* and its value at a time 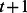 depends on the state of other components of the network at a previous time unit. The state of every node *g* therefore changes according to:
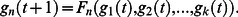
(1)


In this equation, 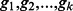 are the regulators of node state 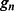 and 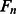 is a discrete function known as a logical rule (Table 3 in File S1; logical rules are grounded in available experimental data). Given the logical rules, it is possible to follow the dynamics of the network for any given initial configuration of the nodes expression state. One of the most important traits of dynamic models is the existence of steady states in which the entire network enters into a self-sustained configuration of the nodes state. It is thought that in developmental systems such self-sustained states correspond to particular cell types [30].

**Figure 3:**
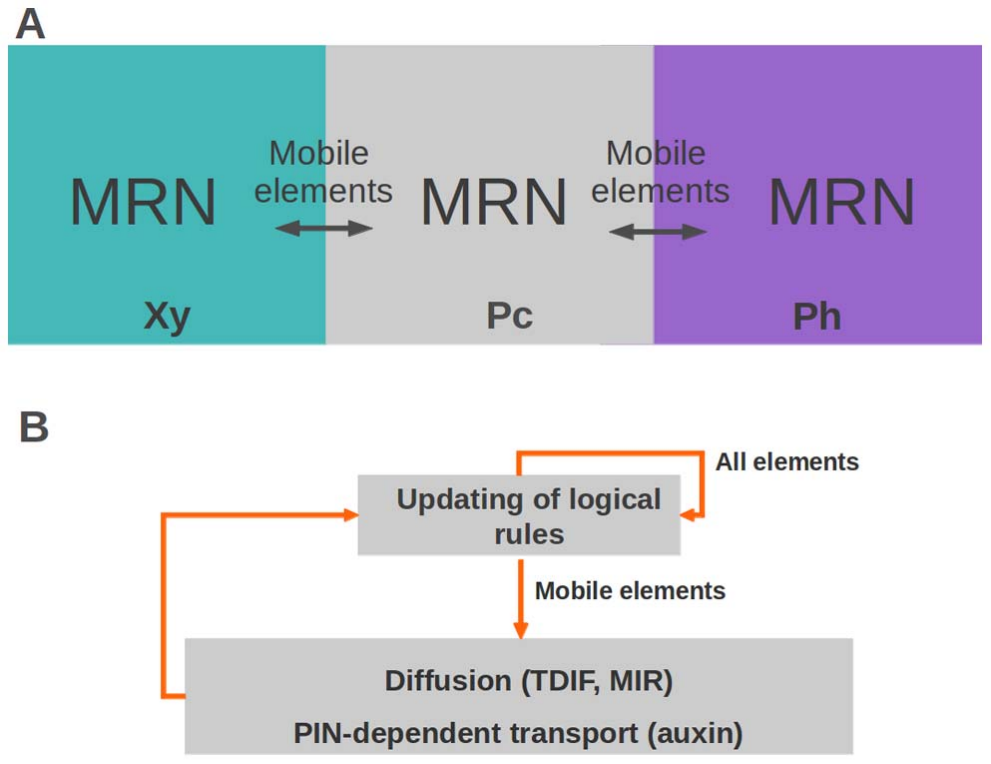
Schematic representation of the spatiotemporal model. (A) Three spatial compartments are considered, each corresponding to a main region of the shoot vascular bundles. Each compartment contains the same molecular regulatory network (MRN) (as specified in Figure 2 and Table 3 of File S1), and certain mobile elements are able to move among cells. (B) In every simulation, the system is started in an initial condition and then the logical rules and the diffusion and transport rules are iteratively applied until a steady state is reached (Methods).

In order to explore the spatiotemporal dynamics of the system under study, we considered three compartments representing the procambial cells and the adjacent young xylem and phloem cells within the shoot vascular bundles (Figure 1). Each cell or compartment has the same regulatory network (Figure 3). However, the networks in each cell are coupled to the neighboring network by means of mobile elements (square-shaped nodes in Figure 2, Figure 3). According to experimental evidence for the system under study (see Table 1 in File S1 for data supporting regulatory interactions and Table 2 in File S1 for a summary of expression and localization patterns), the hormone IAA, the peptide TDIF, and the microRNA MIR165/6 are able to move among the cells. In the case of TDIF and MIR165/6, the mobility is defined as diffusion and is given by the following equation:
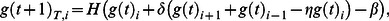
(2)where 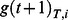 is the total amount of TDIF or MIR165 in cell 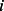. 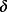 is a parameter that determines the proportion of 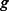 that can move from any cell to neighboring ones and is correlated to the diffusion rate of 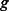. is a constant corresponding to a degradation term. 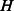 is a step function that converts the continuous values of 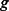 into a discrete variable that may attain values of 0, 1 or 2. 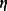 refers to the number of neighboring compartments, which is 2 for the compartment in the procambial position, and 1 for the two compartments located in the extremes of the array (i.e. phloem and xylem). Boundary conditions are zero-flux. In the case of IAA, the mobility is defined as active transport dependent on the radial localization of the PIN efflux transporters (Table 2 in File S1) and is defined by the equation:
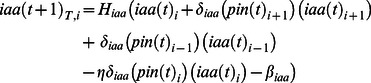
(3)where 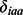 is a parameter that determines the proportion of IAA that can be transported among cells. The transport depends on the presence of IAA and PIN in the cells and 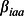 corresponds to a degradation term. As in equation 2, 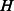 is a step function that converts the continuous values to discrete ones and 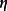 stands for the number of neighbors in each cell. Boundary conditions for IAA motion are also zero-flux.

Using the logical rules (Table 3 in File S1), equations 1–3, and a broad range of parameter values (Table 4 in File S1), it is possible to fully reproduce the results and analyses reported in the following sections (see simulation time course in Figure 1 in File S1). The model was implemented in C.

## Results

### 1 Model Assumptions

In order to focus on the core logic of the shoot vascular patterning network, and given the available experimental evidence, the model incorporates a number of simplifying assumptions. These are as follows:

#### 1.1

The overall structure of the shoot vascular bundles is simplified to a one-dimensional arrangement of three compartments, corresponding to xylem, procambial and phloem cells (Figure 1). Since we find that there is insufficient data to clearly discern steady states expected for the different vascular cell subtypes (e.g. xylem fibers and tracheary elements in xylem), we only consider these three broad types. Although simplifying, this assumption will facilitate future comparative studies with the vascular system at other parts of the plant and even with those of other plants.

#### 1.2

Each of these three cell types has the same regulatory network, but, given the network dynamics, the presence of mobile elements, and the initial conditions, each cell can reach a different state corresponding to a different cell type.

#### 1.3

The state of all nodes in the network is updated simultaneously (synchronous update) in discrete time steps. This is a strong assumption because regulation of the nodes is in fact likely to occur at different rates (see for example [31]). However, given that the data used to build this network were obtained for different moments during plant development, and since there is not enough information to assume a particular sequence for asynchronous update, the synchronous update constitutes the simplest assumption. A consequence of this assumption is that only final steady states (and not transient ones) are informative.

#### 1.4

In order to focus on pattern formation within vascular bundles in the radial direction, cell-to-cell communication and transport in the apical–basal axis is neglected. However, since we focus our study in young provascular (undifferentiated) cells, it is likely that apical–basal transport at this point is still incipient.

#### 1.5

Only those regulatory elements that are involved in the early process of vascular cell specification and patterning are considered, while “downstream” elements acting later in the differentiation process are not included into the model.

### 2 Initial Conditions

The initial conditions specify the initial state of some of the network elements (Figure 1 in File S1) and are the following (a robustness analysis regarding these initial conditions is presented in the sections below):

#### 2.1

In the procambial position (central compartment), CK is initially available and there is an initial and sustained IAA input or self-upregulation. This condition is supported by several lines of evidence [8], [13], [32]–[36]. Also *ATHB-8*, a marker of early vascular development that has been found in preprocambial cells, is assumed to be initially present at this position [19], [37], [38]. These conditions are not fixed, however. After the initial configuration, all the members of the CK and IAA signaling pathways, as well as *ATHB-8*, can change their states according to the logical rules.

#### 2.2

In the xylem and phloem positions, it is assumed that no element is initially active except for the CK signaling pathway and TDIF, both in the phloem position. The localization of TDIF in the phloem is well documented ([26], Table 2 in File S1). At present, however, it is unknown how its spatial pattern is regulated. In our model, therefore, its expression is fixed to the phloem position, although it may move by diffusion during simulations. The localization of CK in the phloem position is given only as an initial condition and can change during simulations according to the dynamic rules (Table 3 in File S1).

### 3 Newly Postulated Interactions

Considering only experimentally verified data for the shoot of Arabidopsis (Figure 2, without considering dotted lines), the model cannot reproduce the expected wild-type configuration of the three basic vascular cell types. In particular, this model is unable to render reported patterns of some of the network elements that may serve as cell-type markers (APL, KAN, VND and ASL) (Table 2 in File S1). This suggests that the reported interactions are necessary but not sufficient for cellular determination and patterning in the shoot vascular bundles. By making a few educated guesses, however, the modified model is able to reproduce them in a robust way. The assumed interactions are represented as dotted lines in Figure 2 and are largely based on the experimental data available for other Arabidopsis tissues, as well as on the reported interactions among genes with similar sequences and functions in other contexts of plant development, particularly the vascular tissue in roots (Tables 1 and 2 in File S1). In the context of our modeling approach, these interactions appear to be necessary for the determination of the basic cell types in the vascular bundles of Arabidopsis, and we therefore postulate these as candidates for experimental validation below.

#### 3.1 CKs upregulate APL

*APL,* a phloem marker, has been reported to be directly or indirectly downregulated by *PXY* [39]. In our model, however, if *APL* is considered to be regulated only by *PXY*, its expression domain also includes the compartment corresponding to young xylem cells. Since this is not observed, we postulate that *APL* is positively regulated by CK signaling (Figure 2, dotted lines). This is consistent with the fact that CKs transported in the phloem are in turn necessary for proper formation of phloem in Arabidopsis root [16]. When the CK-mediated upregulation of *APL* via B-ARRs is included (Figure 2), *APL* is restricted to the phloem. Upregulation of APL by CKs does not contradict the rest of the dataset included in the model and could explain the observed expression patterns. Furthermore, this prediction is supported by the following findings: i) *APL* 5' flanking region has 10 repeats of the CK-responsive (ARR1-dependent) motif (Database of Plant Cis-acting Regulatory DNA Elements, PLACE, [40]), and ii) microarray experiments show a rapid increase in the expression of *APL* after Arabidopsis cell cultures are treated with BAP, a type of CK (TAIR, ExpressionSet:1005823559).

#### 3.2 ATHB-8 and KAN inhibit each other

The complementary distribution of *KAN* and HD ZIP III genes like *ATHB-8* in vascular bundles and other systems suggests that these gene families inhibit each other [41], [42] (Figure 2, dotted lines). The molecular details of such interaction have not been revealed, but it is proposed to be mediated by MIR165/6 [10]. It is not yet understood how the presence of *KAN* is confined to the phloem, but when we consider this mutual downregulation (mediated by MIR165/6, see Figure 2), the model can reproduce the expected cellular profiles, including that of the phloem. In contrast, when the mutually negative regulation between ATHB-8 and KAN is not included into the model, *ATHB-8* and *KAN* oscillate in the procambial and phloem positions, which has not been reported. Alternatively, *KAN* would have to be assumed to be upregulated in the phloem by an unknown factor.

#### 3.3 ATHB-8 self-upregulates via BR

Our model reveals that the reported expression of ATHB-8 in procambial and xylem cells (Table 2 in File S1) can only be robustly reproduced if ATHB-8 is initially present in the procambial cells and its activity is somehow self-sustained. In our simulations, if ATHB-8 is not self-upregulating, its expression in the procambial cells is lost and KAN is ectopically expressed in this position. It was shown that BRs upregulate HD-Zip *III* homeobox genes belonging to the same family as *ATHB-8* [37]. Furthermore, BRs seem to promote procambial identity and transdifferentiation to xylem cells (reviewed in [8]). In our model, therefore, we considered that BRs upregulate *ATHB-8* and that the positive feedback allowing self-sustained upregulation of ATHB-8 is completed by upregulation of BRs by ATHB-8 (Figure 2, dotted lines). This implies a mechanism by which BRs could stabilize *ATHB-8* in procambial and protoxylem cells, as well as the overall xylem–phloem ratios.

#### 3.4 AHP6 is inhibited by CK and upregulated by IAA in the shoot, as it is in the root

These interactions, although probably indirect, have been reported for the root of Arabidopsis [17] but not for the shoot (Figure 2, dotted lines). Nevertheless, it has been shown that *AHP6* is expressed in the shoots and young leaves [17] and, when *AHP6* upregulation by IAA and downregulation by CK is incorporated into our shoot model, the patterns are robustly formed (see also mutant analysis below).

### 4 Robust Specification and Maintenance of the Three Main Vascular Regions in Wild-type Simulations

**Figure 4:**
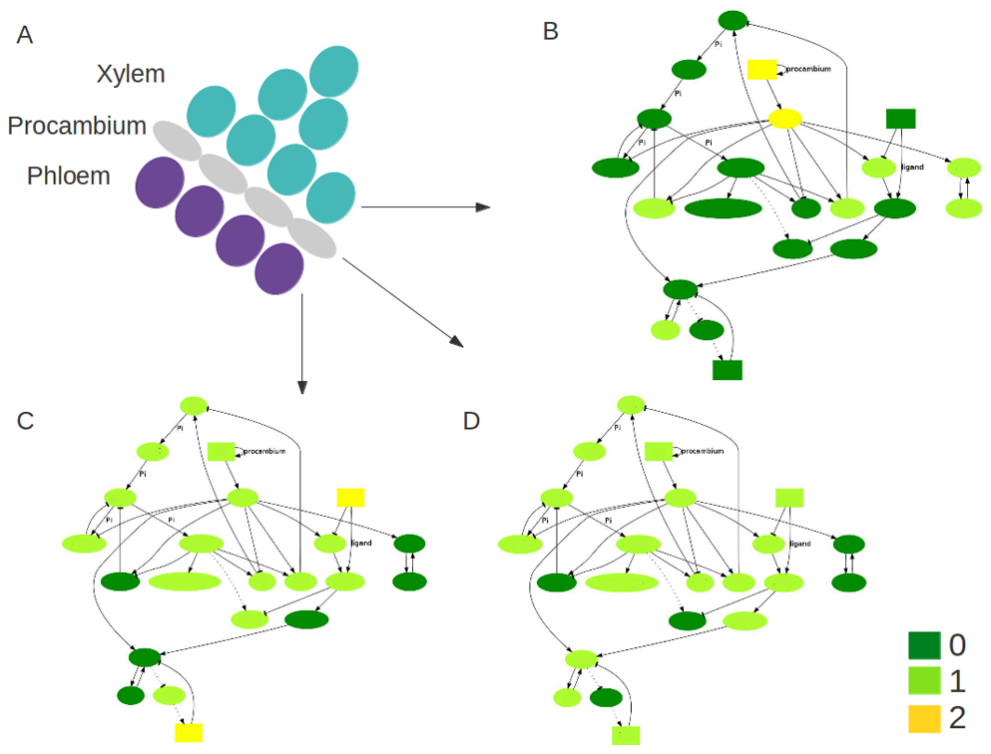
Network steady states characterizing each of the regions within the shoot vascular bundles. (A) Schematic representation of a shoot vascular bundle in Arabidopsis. (B) MRN steady states corresponding to the xylem position, (C) phloem position, and (D) procambium position. The experimentally reported xylem, procambium and phloem markers peak in the corresponding positions. The position of the network nodes corresponds to that in Figure 2.

After the aforementioned postulated interactions were incorporated into the logical rules that specify the model’s dynamics (dotted lines in Figure 2, Table 3 in File S1), the model was able to robustly generate the expected patterns and arrangements (Figure 1). Starting from the initial conditions previously specified, and given the dynamics stipulated in the rules and mobility equations (Table 3 in File S1 and Methods), the elements of the network change their state until the entire system reaches a steady state in which the elements characterizing each vascular region are present. As shown in Figure 4, the steady state reached by the networks within each of the three compartments matches the experimentally reported expression and concentration profiles (Table 2 in File S1). In such final state, *VND* and *ASL* are active only in the xylem position, *WOX4* only in the procambial position, and *APL* only in the phloem position (Figure 1). It is worth noting that this steady state is reached after as few as 5 iterative steps (Figure 1 in File S1).

Furthermore, the steady state of our model exhibits hormonal profiles that are consistent with a mechanism in which IAA and CKs restrain one another’s domains during vascular development [10], [13] and is consistent with expression and metabolic data for the Arabidopsis root vasculature and other plant systems [8], [9], [13] (Table 2 in File S1). As shown in Figure 4, in the steady state reached by the system, IAA peaks in the compartment corresponding to xylem cells, which is in agreement with previous reports ([9], Table 2 in File S1). In the steady state of the simulated system, in turn, CKs are present in procambial and phloem cells.

Since the model could reproduce the expected patterns of these cell-type markers, we then proceeded to test the model’s robustness in the face of its modifications. The model involves some unknown parameters associated with the diffusion and transport of mobile elements (equations 1–3 in Methods). Therefore, we tested whether the overall system was robust to changes in these parameters and found that parameters could be varied in a relatively ample range of values (Table 4 in File S1).

As mentioned above, the model assumes an initial distribution of the hormones IAA and CKs, expression of *ATHB-8*, and localization of TDIF. These initial conditions are in agreement with the available experimental data. It is important, however, to test if other initial conditions can render the wild-type vascular pattern. Additionally, these tests may also point to unknown alterations in the hormonal profiles that could lead to changes in the plant’s vascular pattern. We thus tested how sensitive the system is to changes in each of the aforementioned initial conditions. The system is robust to changes in the initial IAA localization, because, even if IAA is simulated to be initially present in the xylem and phloem positions, the final steady state exhibits an IAA peak in the xylem position, and the expected localization of cell-type markers. On the other hand, changes in CK initial conditions affected the overall pattern generated by the model, not being able to generate the expected patterns when CKs were initially present in the xylem position or when CKs were initially absent from the procambial or phloem positions. We also assume as an initial condition that ATHB-8 is present in the procambial position. Robustness analyses show that when ATHB-8 is not initially present in procambial cells, the overall cellular expression and concentration profiles are conserved. Under these conditions, however, *ATHB-8*, *KAN* and *MIR* oscillate in the cells where they are expressed. Interestingly, their expression is also stabilized in the expected position if BR, which could establish a feedback loop with *ATHB-8* (Figure 2), is assumed to be initially present in the procambial position instead of ATHB-8 [19], [37], [38]. As for the parameter analyses, initial lack of TDIF in the phloem position cannot be accommodated by the system, probably because the additional regulatory inputs that could make it more robust to changes in the expression/localization of TDIF are still unknown.

### 5 Simulation of Hormonal Depletion and Mutant MRNs Supports the Proposed Model

**Figure 5:**
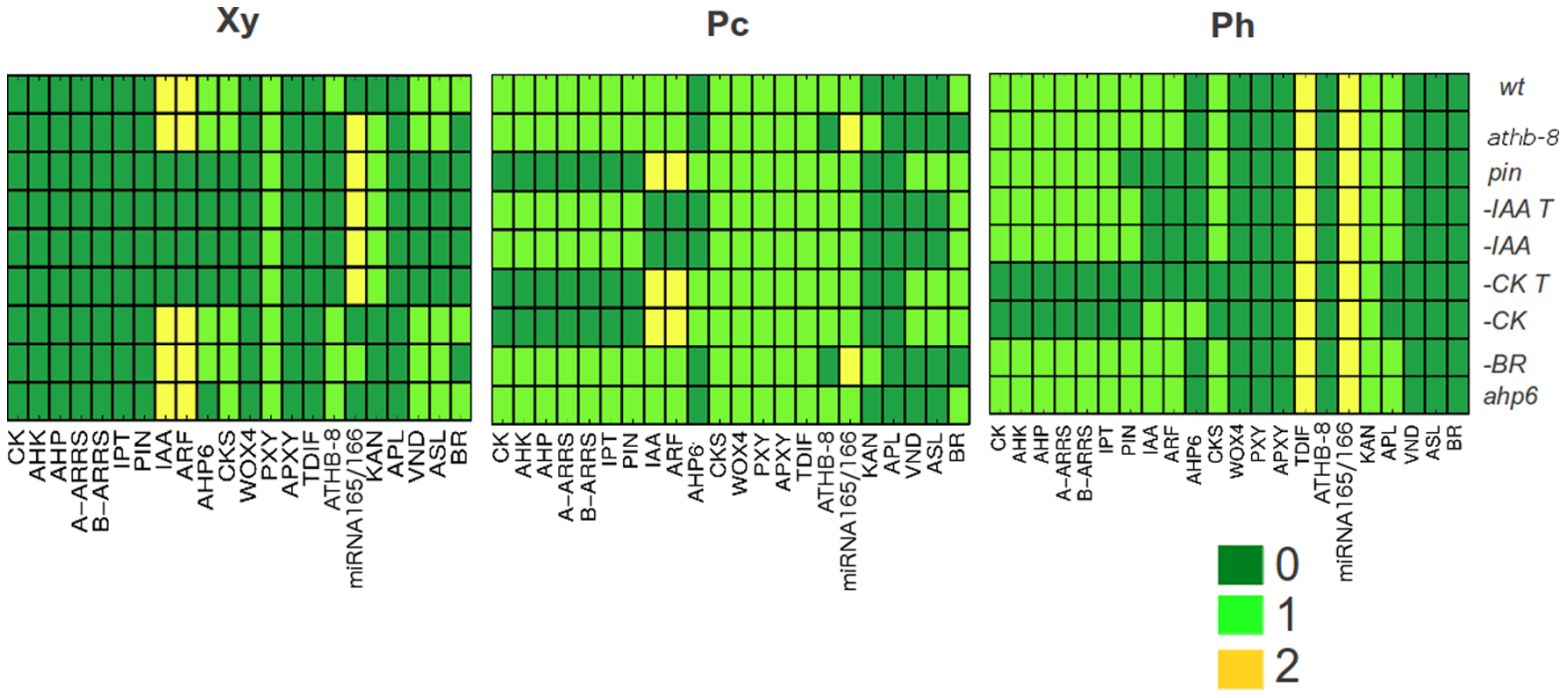
Mutant and hormone depletion simulations using the spatiotemporal network model. Each horizontal line or vector represents the molecular profile of the three tissue types considered in the model. The compartment in the center corresponds to the procambium, while the compartments to the left and right correspond to the xylem and phloem, respectively. The color scale represents the states of the nodes at a given time (0, 1 or 2). Each row corresponds to the final steady profiles of a mutant or hormone-depletion simulation. In *–IAA,* IAA is set to 0 throughout the simulations but is present in the initial conditions. In –IAA *T,* IAA is set to 0 in the initial conditions and throughout the simulations. Likewise, in *−CK,* CK is set to 0 throughout the simulations but is present in the initial conditions. In –CK *T,* CK is set to 0 in the initial conditions and throughout the simulations.

In order to test the model, hormonal-depletion treatments and mutations were simulated and compared with the experimentally reported phenotypes (Figure 5). The particular *in silico* experiments are described below.

#### 5.1 CK depletion

Experimental data show that CK signaling is decreased in the CRE-family triple mutant and the *wol* mutant (gain of function mutation in *AHK4* mimicking the triple CK receptor mutant phenotype [33], [36]), both of which exhibit a lack of phloem and metaxylem, as well as a reduction in the number of vascular cells [10], [17], [33]. A similar phenotype is observed in transgenic lines in which CKs are degraded from the root vascular bundle or soon after the induction of *CKX1*, suggesting that CK signaling primarily inhibits a “default” developmental pathway leading to the xylem specification and promotes procambial cell identity [17]. In the shoot, mutants in which CK signaling is decreased also exhibit fewer vascular cells but do not lose phloem cells completely [15]. In agreement with these lines of evidence, when we use our model to simulate total CK depletion (*–CK T* in Figure 5, CK value is set to 0 in the initial conditions and throughout simulations) or partial CK depletion (*–CK* in Figure 5, CK is set to 0 throughout simulations but is present in the initial conditions), the xylem markers *VND* and *ASL* are found in the procambium position. This suggests that CK depletion could delimit the mitotic activity or identity of stem cells, leading to progressive xylem differentiation. As a consequence, consuming the pool of stem cells in the meristem would result in a reduced amount of vascular cells. When total CK depletion is modeled in our system, moreover, *APL* is no longer expressed in the phloem position. This is consistent with the phloem-less *wol* phenotype of the root [33].

#### 5.2 IAA depletion

Several lines of empirical data show that IAA depletion prevents xylem differentiation [10], [13], [44]. This is well reproduced in the IAA depletion simulations performed here. As shown in Figure 5, both partial IAA depletion (*–IAA* in Figure 5, IAA is set to 0 throughout simulations but is present in the initial conditions) and total IAA depletion (*–IAA T* in Figure 5, IAA value is set to 0 in the initial conditions and throughout simulations) result in a lack of expression or accumulation of xylem markers (VND and ASL), suggesting the loss of xylem cell identity.

#### 5.3 BR depletion

It has been experimentally shown in Zinnia that BRs facilitate the differentiation of mechanically isolated mesophyll cells into xylem cells, possibly through the regulation of specific members of the ZeHD-ZIP III family [8], [37]. Moreover, BRs appear to be necessary for the transdifferentiation of Arabidopsis hypocotyl cells into xylem cells [45]. Indeed, when the BR receptor *BRL1* is mutated, plants display increased phloem and reduced xylem differentiation [46]. In our model, the simulation of BR depletion causes a loss of *ATHB-8* in the procambial position and consequent ectopic expression of *KAN* in the procambium (*-BR* in Figure 5). Since *KAN* is mainly expressed in the phloem (Table 2 in File S1) and HD-ZIP III genes (including *ATHB-8*) are mainly expressed in procambium and xylem cells, this simulation result could correspond to the commitment of more procambial cells into phloem than into xylem. That corresponds well with the observed *brl1* phenotype [46].

#### 5.4 pin mutant

Experimentally, the application of 1-N-naphthylphthalamic acid (NPA), an inhibitor of polar auxin transport (PAT), prevents TE differentiation in Zinnia [47]. The shoots of PAT-defective Arabidopsis plants (both NPA-treated plants and mutants in IAA efflux carriers from the PIN family) reveal an abnormal number of vascular bundles and xylem proliferation [48]. In the roots of Arabidopsis, application of NPA also suppresses protoxylem formation [13]. Double knock-out mutants in IAA efflux carriers *PIN3* and *PIN7* form unstable IAA patterns. In some individuals, the IAA response maxima are expanded and plants have roots with ectopic protoxylem, while in others the maxima are reduced and plants show loss of protoxylem compared to wild-type [11]. We observe in our simulations the latter type of phenotypic aberrations, in which cells in the xylem position lack the presence of xylem markers VND and ASL (*pin* in Figure 5).

#### 5.5 athb-8 mutant

Literature reports indicate that when all five HD-ZIP III (including *ATHB-8*) are knocked out, xylem formation is no longer detected in the root and it is reduced in the shoot [49], [50]. In the computational simulation of the *athb-8* mutant, we observe that some of the xylem-characteristic elements are still present in the xylem positions but that phloem elements (KAN and MIR) are also expressed there, thus rendering a mis-specified xylem cell (*athb-8* in Figure 5). Since we are considering the collective and partially redundant action of HD-ZIP III genes in the ATHB-8 node, our results match the experimental data. Additionally, even in the multiple HD-ZIP III mutant, phloem cells are still formed [50]. That corresponds with our simulations, as the phloem profile is still reproduced in the corresponding position (*athb-8* in Figure 5).

#### 5.6 ahp6 mutant

The *ahp6* mutant seedlings of Arabidopsis display no obvious phenotype at the macroscopic level. Sporadic protoxylem differentiation is observed, however, along the root (i.e. in some sections of the root one of the two protoxylem cell files is absent). This phenotype could be mimicked by addition of exogenous CKs with *ahp6* roots being more sensitive to CK-mediated loss of protoxylem cell identity, which is consistent with the role of *AHP6* as a negative regulator of CK signaling [17]. In our model, the simulated loss of *ahp6* function results in no alteration as compared to the wild-type vascular arrangement (*ahp6* in Figure 5). However, the *ahp6* mutant version of the model cannot buffer the simulated addition of CKs into the xylem position, thus rendering altered patterns. In contrast, the wild-type version of the model, in which *AHP6* is active, can still give rise to the wild-type pattern and profiles even when CKs are “added” to the xylem position during the simulations. This is consistent with the aforementioned increased sensibility to CKs reported in the roots of *ahp6* mutant plants [17]. Although the interactions regulating vascular patterning are probably different in the root and shoot of Arabidopsis, our results suggest that *AHP6* has a similar role in the two types of vascular tissue. Thus, a possible explanation for the *ahp6* phenotype observed in the root may be that this mutant cannot buffer spontaneous fluctuations in the abundance of CKs.

Overall, the aforementioned simulation results correspond closely to published data and may provide further insights into the mechanisms leading to certain mutant phenotypes that have been reported.

### 6 Vascular Patterning Systems in Zinnia and Arabidopsis are Dynamically Similar

Single mesophyll cells of Zinnia that have been isolated and cultured *in vitro* with added CKs and auxin (although 1-Naphthaleneacetic acid, was used instead of IAA) transdifferentiate at a high frequency into xylem cells. It has been shown that this process requires the transport of auxin (both into and out of the cells) and that brassinosteroids are synthesized actively during the differentiation to xylem cells [8], [43], [44], [51]. In order to test whether the described molecular mechanisms are similar – at the dynamic level – in other plants, and thus whether our model could be used for further comparative studies, we used its modified version to simulate the dynamics of a single (Zinnia) cell in a medium with relatively high levels of CKs and auxin (as originally reported by Fukuda and Komamine [52]). In this case, we modeled only one compartment with the initial conditions that we have used for the procambial position: IAA, assuming it acts like NAA, and CK signaling systems, as well as *ATHB-8* activated. Since individual Zinnia cells are isolated in the reported experiments, we neglected intercellular communication. As in the reported experiments, the modeled cell was considered to sense CKs and transport IAA from and to the medium (equations 2 and 3 in Methods). The model shows that from this initial condition, and in an isolated single-cell system, the collective action of genes and hormones leads to a steady state in which a xylem profile is achieved (data not shown). Also, as has been experimentally reported, brassinosteroids are upregulated during the simulation in our model. This suggests that even if the molecular regulatory network involved in the specification of vascular types of cells in *Zinnia* were different from that acting in *Arabidopsis,* it is likely that the overall topology and dynamics of these networks are similar, which is also supported by the participation of genes from the same families in the vascular patterning of both plant species [8]. This result suggests that our model is a valuable tool for performing comparative studies at the dynamic level and supports the hypothesis stating that protoxylem is the “default” vascular identity [17].

## Discussion

Given certain simplifying assumptions and a few newly proposed interactions (unverified but based on related data; Tables 1 and 2 in File S1), the presented model can account for the wild-type organization of vascular bundles, as well as for some qualitative mutant phenotypes (e.g. complete loss of a cell type) and hormone depletion phenotypes, thus providing a plausible dynamic mechanism for cellular determination and patterning in the vascular bundles of Arabidopsis. Moreover, the model has led to the postulation of new interactions that may be experimentally tested (dotted lines in Figure 2).

### 1 Differences in Hormonal Control Over Primary and Secondary Growth

As shown in the Results section, our model supports a mechanism in which IAA and CKs restrain each other’s domains during vascular development [10], [13]. According to the model results, IAA peaks in xylem cells and CKs are present only in the phloem and procambial positions, where IAA is present just in low levels. Suer et al. [53] analyzed the IAA reporter DR5rev:GFP along the cambium at the onset and during secondary thickening of the inflorescence stem. They found the IAA maxima located mainly in the phloem cells (both phloem of vascular bundles and secondary phloem in the interfascicular regions) and in single cortex cells of interfascicular arcs. These results contrast with our model and with the previously reported results showing a peak of DR5::GUS in the procambial and differentiating xylem in the primary vasculature [11]. Thus, if present, phloem-located IAA could have been below the detection levels. Our model, however, is intended to explain cell-fate determination in the proximity of the procambial cells and early during primary vascular development and patterning. Thus, the aforementioned experimentally reported differences in IAA distribution point to possible specificity in hormonal regulation over primary and secondary growth.

### 2 Model Limitations

The results presented in this work can be reproduced by implementing the logical rules (Table 3 in File S1) and equations presented in the Methods section within the parameter ranges specified in Table 4 in File S1. Therefore, we expect that it will provide the scientific community with a useful framework to continue integrating the data emerging for the system under study, as well as for testing hypotheses *in silico* and restraining future experiments. However, the methods and simplifications used to implement this model convey certain limitations that must be considered. First, individual cells cannot be resolved within the three model compartments (representing phloem, xylem and procambium), thus cell proliferation and differentiation of individual cell subtypes (e.g. tracheary elements and xylem fibers in the xylem) cannot be considered in the model. Further, since the compartments have a fixed size, our model cannot reproduce changes in the relative size of the vascular tissues or number of cell lines. Also, the model does not at this point account for apical–basal variations in the vascular patterns. Finally, robustness analyses reveal that the model is particularly sensitive to certain changes in the initial conditions, and especially in the initial profile of CKs. This lack of robustness in the case of certain initial conditions may reflect that additional interactions are yet to be incorporated or that there are incorrect assumptions to be experimentally rejected. Alternatively, the sensitivity to initial CK content may reflect a central role of CKs in vascular patterning, as is also suggested by the strong vascular phenotype of the *wol* mutant in Arabidopsis root [33]. The critical role of CKs is further supported by the presence of several mechanisms bypassing fluctuations in CK content and buffering the effect of partial absence of CK signaling or biosynthesis (e.g. increase in CK biosynthesis in the multiple CK signaling mutants) [54].

Also worthy of note is that in the current version of our model we consider CKs to be the only input to the MSP signaling system. Although the MSP system does seem primarily to mediate CK action, it nevertheless has been shown that a number of other hormones and signals converge at the MSP. For example, CKs establish crosstalk with ethylene and salicylic acid at the level of MSP components [55]–[57] and light also appears to interfere with the CK response by means of interactions between phytochromes and MSP [56]–[60]. In addition, MSP seems to integrate further signals like CK-independent CKI1 activity, osmoregulation or abscisic acid activity (reviewed in [61]). At present, we consider there to be insufficient data to include these pathways into the model, although their inclusion into future versions could help in understanding the plastic nature of vascular development in response to environmental and organismal stimuli. Importantly, integrating other inputs into MSP could fill the proposed gap in the model and reduce its sensitivity concerning CK initial conditions and fluctuations.

### 3 Future Outlooks

As more information becomes available, it also will be possible to study the differentiation of cellular or tissue subtypes (such as the different types of xylem cells). It has been shown, for example, that MIR165/6 is important for determining the different types of xylem cells (metaxylem and protoxylem) in the root [62], and future developments of this model could help in understanding the dynamics of such sub-differentiation. Similarly, it might be possible to include more information and dispense with certain model assumptions regarding, for example, the initial conditions, hormonal crosstalk, and precise molecular nature of some of those interactions considered here. In turn, we expect that this integrative effort will help in designing future experiments. In particular, we suggest that it would be of great interest to test experimentally those interactions that are heretofore merely postulated (dotted lines in Figure 2).

Understanding the processes behind plastic development is central to comprehending the origin and evolution of an organism’s forms [63], [64]. In particular, studying the dynamics of vascular patterning will help clarify how lateral growth in plants responds to different types of stimuli and how these stimuli are integrated and translated into patterns of specific tissues during postembryonic development. Moreover, a better understanding of how vascular tissues are patterned and organized will enable performing comparative analyses to elucidate the general mechanisms driving cell division and cell differentiation in plants and even in other multicellular organisms [65], [29]. The molecular elements involved in determining vascular tissues in the inflorescence stem are partially shared with, or belong to the same families as, those involved in similar processes in the roots, leaves, and both apical (shoot and root) meristems of Arabidopsis (e.g. [13], [53], [66]). Thus, the use of dynamic models will allow for testing if there is a “dynamic core” shared by the vascular patterning in various organs and other tissue patterning systems, particularly apical meristems, even if there are tissue-specific elements or interactions. Along that same line, dynamic and integrative models could complement the comparative studies being performed to examine vascular patterning in different plants and eventually shed light on the origin and variation of vascular arrangements and the evolution of plants’ vascular patterning.

## Supporting Information

File S1 **Supporting file including tables with the experimental data on which the model is based, the dynamical rules to implement the model, details of the robustness analysis, and a supporting figure illustrating a realization of the computational model.**
(DOC)

## References

[1] Azpeitia E, Benítez M, Vega I, Villarreal C, Alvarez-Buylla ER (2010) Single-cell and coupled GRN models of cell patterning in the Arabidopsis thaliana root stem cell niche. BMC Syst Biol 4: 134. 20920363 10.1186/1752-0509-4-134 PMC2972269

[2] Fujita H, Toyokura K, Okada K, Kawaguchi M (2011) Reaction-diffusion pattern in shoot apical meristem of plants. PloS One 6: e18243. 21479227 10.1371/journal.pone.0018243 PMC3066213

[3] Vernoux T, Brunoud G, Farcot E, Morin V, Van den Daele H, et al (2011) The auxin signalling network translates dynamic input into robust patterning at the shoot apex. Mol Syst Biol 7: 508. 21734647 10.1038/msb.2011.39 PMC3167386

[4] Turner S, Sieburth LE (2003) Vascular patterning. In: The Arabidopsis Book. American Society of Plant Biologists 2: e0073. 10.1199/tab.0073 PMC3243335 22303224

[5] Esau K (1977) Anatomy Of Seed Plants. John Wiley & Sons. 576 p.

[6] Altamura MM, Possenti M, Matteucci A, Baima S, Ruberti I, et al (2001) Development of the vascular system in the inflorescence stem of *Arabidopsis* . New Phytol 151: 381–389.

[7] Lehesranta SJ, Lichtenberger R, Helariutta Y (2010) Cell-to-cell communication in vascular morphogenesis. Curr Op Plant Biol 13: 59–65. 10.1016/j.pbi.2009.09.004 19783199

[8] Fukuda H (2004) Signals that control plant vascular cell differentiation. Nat Rev Mol Cell Biol 5: 379–391. 15122351 10.1038/nrm1364

[9] Elo A, Immanen J, Nieminen K, Helariutta Y (2009) Stem cell function during plant vascular development.Semin Cell Dev Biol. 20: 1097–1106. 10.1016/j.semcdb.2009.09.009 19770063

[10] Caño-Delgado A, Lee JY, Demura T (2010) Regulatory mechanisms for specification and patterning of plant vascular tissues. Ann Rev Cell Dev Biol 26: 605–637. 20590454 10.1146/annurev-cellbio-100109-104107

[11] Ibañes M, Fàbregas N, Chory J, Caño-Delgado AI (2009) Brassinosteroid signaling and auxin transport are required to establish the periodic pattern of Arabidopsis shoot vascular bundles. Proc Nat Acad Sci USA 106: 13630–13635. 19666540 10.1073/pnas.0906416106 PMC2717112

[12] De Smet I, Tetsumura T, De Rybel B, Frey NFD, Laplaze L, et al (2007) Auxin-dependent regulation of lateral root positioning in the basal meristem of Arabidopsis. Development 134: 681–690. 17215297 10.1242/dev.02753

[13] Bishopp A, Help H, El-Showk S, Weijers D, Scheres B, et al (2011) A mutually inhibitory interaction between auxin and cytokinin specifies vascular pattern in roots. Curr Biol 21: 917–926. 21620702 10.1016/j.cub.2011.04.017

[14] Matsumoto-Kitano M, Kusumoto T, Tarkowski P, Kinoshita-Tsujimura K, Václavíková K, et al (2008) Cytokinins are central regulators of cambial activity. Proc Nat Acad Sci USA 105: 20027–20031. 19074290 10.1073/pnas.0805619105 PMC2605004

[15] Hejátko J, Ryu H, Kim GT, Dobesová R, Choi S, et al (2009) The histidine kinases CYTOKININ-INDEPENDENT1 and ARABIDOPSIS HISTIDINE KINASE2 and 3 regulate vascular tissue development in Arabidopsis shoots. Plant Cell 21: 2008–2021. 19622803 10.1105/tpc.109.066696 PMC2729606

[16] Bishopp A, Lehesranta S, Vatén A, Help H, El-Showk S, et al (2011) Phloem-transported cytokinin regulates polar auxin transport and maintains vascular pattern in the root meristem. Curr Biol 21: 927–932. 21620705 10.1016/j.cub.2011.04.049

[17] Mahönen AP, Bishopp A, Higuchi M, Nieminen KM, Kinoshita K, et al (2006) Cytokinin signaling and its inhibitor AHP6 regulate cell fate during vascular development. Science 311: 94–98. 16400151 10.1126/science.1118875

[18] Hwang I, Sheen J, Müller B (2012) Cytokinin signaling networks. Ann Rev Plant Biol 63: 353–380. 22554243 10.1146/annurev-arplant-042811-105503

[19] Baima S, Nobili F, Sessa G, Lucchetti S, Ruberti I, et al (1995) The expression of the Athb-8 homeobox gene is restricted to provascular cells in Arabidopsis thaliana. Development 121: 4171–4182. 8575317 10.1242/dev.121.12.4171

[20] Baima S, Possenti M, Matteucci A, Wisman E, Altamura MM, et al (2001) The Arabidopsis ATHB-8 HD-zip protein acts as a differentiation-promoting transcription factor of the vascular meristems. Plant Physiol 126: 643–655. 11402194 10.1104/pp.126.2.643 PMC111156

[21] Kubo M, Udagawa M, Nishikubo N, Horiguchi G, Yamaguchi M, et al (2005) Transcription switches for protoxylem and metaxylem vessel formation. Genes Dev 19: 1855–1860. 16103214 10.1101/gad.1331305 PMC1186185

[22] Yamaguchi M, Kubo M, Fukuda H, Demura T (2008) Vascular-related NAC-DOMAIN7 is involved in the differentiation of all types of xylem vessels in Arabidopsis roots and shoots. Plant J 55: 652–664. 18445131 10.1111/j.1365-313X.2008.03533.x

[23] Bonke M, Thitamadee S, Mähönen AP, Hauser M, Helariutta Y (2003) APL regulates vascular tissue identity in Arabidopsis. Nature 426: 181–186. 14614507 10.1038/nature02100

[24] Ji J, Shimizu R, Sinha N, Scanlon MJ (2010) Analyses of WOX4 transgenics provide further evidence for the evolution of the WOX gene family during the regulation of diverse stem cell functions. Plant Signal Behav 5: 916–920. 20495368 10.4161/psb.5.7.12104 PMC3014546

[25] Fisher K, Turner S (2007) PXY, a receptor-like kinase essential for maintaining polarity during plant vascular-tissue development. Curr Biol 17: 1061–1066. 17570668 10.1016/j.cub.2007.05.049

[26] Etchells JP, Turner SR (2010) The PXY-CLE41 receptor ligand pair defines a multifunctional pathway that controls the rate and orientation of vascular cell division. Development 137: 767–774. 20147378 10.1242/dev.044941

[27] Ohashi-Ito K, Fukuda H (2010) Transcriptional regulation of vascular cell fates. Curr Op Plant Biol 13: 670–676. 10.1016/j.pbi.2010.08.011 20869293

[28] La Rota C, Chopard J, Das P, Paindavoine S, Rozier F, et al (2011) A Data-Driven Integrative Model of Sepal Primordium Polarity in Arabidopsis. Plant Cell 23: 1–17. 10.1105/tpc.111.092619 PMC3269868 22198150

[29] Hernández-Hernández V, Niklas KJ, Newman SA, Benítez M (2012) Dynamical patterning modules in plant development and evolution. Int J Dev Biol 56: 661–74. 23319343 10.1387/ijdb.120027mb

[30] Kauffman SA (1969) Metabolic stability and epigenesis in randomly constructed genetic nets. J Theor Biol 22: 437–467. 5803332 10.1016/0022-5193(69)90015-0

[31] Sankar M, Osmont KS, Rolcik J, Gujas B, Tarkowska D, et al (2011) A qualitative continuous model of cellular auxin and brassinosteroid signaling and their crosstalk. Bioinformatics 27: 1404–1412. 21450717 10.1093/bioinformatics/btr158

[32] Okadalat K, Uedalb J, Komaki MK, Bell CJ (1991) Requirement of the Auxin Polar Transport System in Early Stages of Arabídopsis Floral Bud Formation. Plant Cell 3: 677–684. 12324609 10.1105/tpc.3.7.677 PMC160035

[33] Mähönen AP, Bonke M, Kauppinen L, Riikonen M, Benfey PN, et al (2000) A novel two-component hybrid molecule regulates vascular morphogenesis of the Arabidopsis root. Genes Dev 14: 2938–43. 11114883 10.1101/gad.189200 PMC317089

[34] Mattsson J, Sung ZR, Berleth T (1999) Responses of plant vascular systems to auxin transport inhibition. Development 126: 2979–2991. 10357941 10.1242/dev.126.13.2979

[35] Turner S, Sieburth LE (2002) Vascular Patterning. In The Arabidopsis Book. American Society of Plant Biologists 5: 1. 10.1199/tab.0073 PMC3243335 22303224

[36] Mähönen AP, Higuchi M, Törmäkangas K, Miyawaki K, Pischke MS, et al (2006) Cytokinins regulate a bidirectional phosphorelay network in Arabidopsis. Curr Biol 1611: 1116–22. 10.1016/j.cub.2006.04.030 16753566

[37] Ohashi-Ito K, Fukuda H (2003) HD-zip III homeobox genes that include a novel member, ZeHB-13 (Zinnia)/ATHB-15 (Arabidopsis), are involved in procambium and xylem cell differentiation. Plant Cell Physiol 44: 1350–1358. 14701930 10.1093/pcp/pcg164

[38] Gardiner J, Donner TJ, Scarpella E (2011) Simultaneous activation of SHR and ATHB8 expression defines switch to preprocambial cell state in Arabidopsis leaf development. Dev Dyn 240: 261–270. 21128301 10.1002/dvdy.22516

[39] Whitford R, Fernandez A, De Groodt R, Ortega E, Hilson P (2008) Plant CLE peptides from two distinct functional classes synergistically induce division of vascular cells. Proc Nat Acad Sci USA 105: 18625–18630. 19011104 10.1073/pnas.0809395105 PMC2587568

[40] Higo K, Ugawa Y, Iwamoto M, Korenaga T (1999) Plant cis-acting regulatory DNA elements (PLACE) database. Nucleic Acids Res 27: 297–300. 9847208 10.1093/nar/27.1.297 PMC148163

[41] Emery JF, Floyd SK, Alvarez J, Eshed Y, Hawker NP, et al (2003) Radial Patterning of Arabidopsis Shoots by Class III HD-ZIP and KANADI Genes. Curr Biol 13: 1768–1774. 14561401 10.1016/j.cub.2003.09.035

[42] Ilegems M, Douet V, Meylan-Bettex M, Uyttewaal M, Brand L, et al (2010) Interplay of auxin, KANADI and Class III HD-ZIP transcription factors in vascular tissue formation. Development 137: 975–984. 20179097 10.1242/dev.047662

[43] Demura T, Tashiro G, Horiguchi G, Kishimoto N, Kubo M, et al (2002) Visualization by comprehensive microarray analysis of gene expression programs during transdifferentiation of mesophyll cells into xylem cells. Proc Nat Acad Sci USA 99: 15794–15799. 12438691 10.1073/pnas.232590499 PMC137795

[44] Yoshida S, Iwamoto K, Demura T, Fukuda H (2009) Comprehensive analysis of the regulatory roles of auxin in early transdifferentiation into xylem cells. Plant Mol Biol 70: 457–469. 19326244 10.1007/s11103-009-9485-y

[45] Sawa S, Demura T, Horiguchi G, Kubo M, Fukuda H (2005) The ATE Genes Are Responsible for Repression of Transdifferentiation into Xylem Cells in Arabidopsis. Plant Physiol 137: 141–148. 15618413 10.1104/pp.104.055145 PMC548845

[46] Caño-Delgado A, Yin Y, Yu C, Vafeados D, Mora-García S, et al (2004) BRL1 and BRL3 are novel brassinosteroid receptors that function in vascular differentiation in Arabidopsis. Development 131: 5341–5351. 15486337 10.1242/dev.01403

[47] Yoshida S, Kuriyama H, Fukuda H (2005) Inhibition of transdifferentiation into tracheary elements by polar auxin transport inhibitors through intracellular auxin depletion. Plant Cell Physiol 46: 2019–2028. 16230330 10.1093/pcp/pci217

[48] Gälweiler L, Guan C, Müller A, Wisman E, Mendgen K, et al (1998) Regulation of polar auxin transport by AtPIN1 in Arabidopsis vascular tissue. Science 282: 2226–30. 9856939 10.1126/science.282.5397.2226

[49] Prigge MJ, Otsuga D (2005) Class III Homeodomain-Leucine Zipper Gene Family Members Have Overlapping, Antagonistic, and Distinct Roles in Arabidopsis Development. 17: 61–76. 10.1105/tpc.104.026161 PMC544490 15598805

[50] Carlsbecker A, Helariutta Y (2005) Phloem and xylem specification: pieces of the puzzle emerge. Curr Op Plant Biol 8: 512–517. 10.1016/j.pbi.2005.07.001 16039153

[51] Fukuda H, Komamine A (1980) Direct Evidence for Cytodifferentiation to Tracheary Elements without Intervening Mitosis in a Culture of Single Cells Isolated from the Mesophyll of Zinnia elegans. Plant Physiol 65: 61–64. 16661144 10.1104/pp.65.1.61 PMC440266

[52] Fukuda H, Komamine A (1980) Establishment of an Experimental System for the Study of Tracheary Element Differentiation from Single Cells Isolated from the Mesophyll of Zinnia elegans. Plant Physiol 65: 57–60. 16661142 10.1104/pp.65.1.57 PMC440265

[53] Suer S, Agusti J, Sanchez P, Schwarz M, Greb T (2011) WOX4 Imparts Auxin Responsiveness to Cambium Cells in Arabidopsis. Plant Cell 23: 3247–3259. 21926336 10.1105/tpc.111.087874 PMC3203433

[54] Riefler M, Novak O, Strnad M, Schmülling T (2006) Arabidopsis cytokinin receptor mutants reveal functions in shoot growth, leaf senescence, seed size, germination, root development, and cytokinin metabolism. Plant Cell 18(1): 40–54. 16361392 10.1105/tpc.105.037796 PMC1323483

[55] Hass C, Lohrmann J, Albrecht V, Sweere U, Hummel F, et al (2004) The response regulator 2 mediates ethylene signalling and hormone signal integration in Arabidopsis. EMBO J 23: 3290–3302. 15282545 10.1038/sj.emboj.7600337 PMC514511

[56] Kushwah S, Jones AM, Laxmi A (2011) Cytokinin interplay with ethylene, auxin and glucose signaling controls Arabidopsis seedling root directional growth. Plant Physiol 156: 1851–1866. 21666052 10.1104/pp.111.175794 PMC3149928

[57] Argueso CT, Ferreira FJ, Epple P, To JPC, Hutchison CE, et al (2012) Two-component elements mediate interactions between cytokinin and salicylic acid in plant immunity. PLoS Genet 8: e1002448. 22291601 10.1371/journal.pgen.1002448 PMC3266875

[58] Sweere U, Eichenberg K, Lohrmann J, Mira-Rodado V, Bäurle I, et al (2001) Interaction of the response regulator ARR4 with phytochrome B in modulating red light signaling. Science 294: 1108–1111. 11691995 10.1126/science.1065022

[59] Mira-Rodado V, Sweere U, Grefen C, Kunkel T, Fejes E, et al (2007) Functional cross-talk between two-component and phytochrome B signal transduction in Arabidopsis. J Exp Bot 58: 2595–2607. 17545225 10.1093/jxb/erm087

[60] Yoshida S, Mandel T, Kuhlemeier C (2011) Stem cell activation by light guides plant organogenesis. Genes Dev 25: 1439–1450. 21724835 10.1101/gad.631211 PMC3134086

[61] Horák J, Janda L, Pekárová B, Hejátko J (2011) Molecular mechanisms of signalling specificity via phosphorelay pathways in Arabidopsis. Curr Protein Pept Sci 12: 126–36. 21348845 10.2174/138920311795684940

[62] Carlsbecker A, Lee JY, Roberts CJ, Dettmer J, Lehesranta S, et al (2010) Cell signalling by microRNA165/6 directs gene dose-dependent root cell fate. Nature 465: 316–321. 20410882 10.1038/nature08977 PMC2967782

[63] Müller GB (2007) Evo-devo: extending the evolutionary synthesis. Nat Rev Genet 8: 943–949. 17984972 10.1038/nrg2219

[64] Finet C, Jaillais Y (2012) Auxology: when auxin meets plant evo-devo. Dev Biol 369: 19–31. 22687750 10.1016/j.ydbio.2012.05.039

[65] Meyerowitz EM (2002) Plants compared to animals: The broadest comparative study of development. Science 295: 1482–1485. 11859185 10.1126/science.1066609

[66] Nakata M, Matsumoto N, Tsugeki R, Rikirsch E, Laux T, et al (2012) Roles of the Middle Domain – Specific WUSCHEL-RERELATED HOMEOBOX genes in early development of leaves in Arabidopsis. Plant Cell 24: 519–35. 22374393 10.1105/tpc.111.092858 PMC3315230

