# Supplementary material for: Dynamics of Cell-Fate Determination and Patterning in the Vascular Bundles of Arabidopsis thaliana
Source: PLoS One. 2013 May 27;8(5):e63108. doi: 10.1371/journal.pone.0063108 (PMC3664626; doi:10.1371/journal.pone.0063108)
Supplement: File S1 — Supporting file including tables with the experimental data on which the model is based, the dynamical rules to implement the model, details of the robustness analysis, and a supporting figure illustrating a realization of the computational model. (DOC) [file pone.0063108.s001.doc]

**Supporting information for “Dynamics of cell-fate determination and patterning in the vascular bundles of *Arabidopsis thaliana”***

Mariana Benítez^1,2^ and Jan Hejátko^1^*

**^1^** Functional Genomics and Proteomics of Plants, Central European Institute of Technology, Masaryk University, Brno, Czech Republic

^2^ Departamento de Ecología de la Biodiversidad, Instituto de Ecología, Universidad Nacional Autónoma de México, , México DF, MEXICO.

*E-mail: jan.hejatko@ceitec.muni.cz

**Table 1. Summary of experimental evidence supporting the model interactions.** Newly postulated interactions are highlighted in gray.

| **Interaction** | **Evidence** | **References** |
| --- | --- | --- |
| A-ARRs –| CK signaling | Double and higher order type-A ARR mutants show increased sensitivity to CK.  Spatial patterns of A-type ARR gene expression and CK response are consistent with partially redundant function of these genes in CK signaling.  A-type ARRs decreases B-type ARR6-LUC.  Note: In certain contexts, however, some A-ARRs appear to have effects antagonistic to other A-ARRs. | [27]⁠  [27]⁠  [13]⁠  [27]⁠ |
| AHP6 –| AHP | *ahp6* partially recovers the mutant phenotype of the CK receptor *WOL*.  Using an in vitro phosphotransfer system, it was shown that, unlike the AHPs, native AHP6 was unable to accept a phosphoryl group. Nevertheless, AHP6 is able to inhibit phosphotransfer from other AHPs to ARRs. | [9]⁠  [9]⁠ |
| AHPs → A-ARRs  (as part of the MSP[[1]](#footnote-2)) | ARR6-LUC is specifically induced by CK.  The expression of ARR3 to ARR9 is induced very rapidly and specifically by cytokinin.  AHPs act as positive regulators of MSP-mediated CK signaling.  Treatment with exogenous cytokinin activated ARR5::GUS expression ubiquitously.  AHP1-GFP and AHP2-GFP seem to move from cytoplasm to nucleus after treatment with CK. However, recent results suggest that AHP traffic within the cell is CK-independent and that, instead, AHP proteins are constantly cycling between the nucleus and cytosol.  Cytokinin treatment increased RNA for the Type-A ARR.  Analogous with two-component systems in other organisms. | [13]⁠  [17]⁠  [78]  [17]⁠    [13,53]⁠  [50–52]⁠  [13]⁠ |
| AHPs → B-ARRs  (as part of the MSP) | AHP1, AHP2, and AHP3 interact with ARR1 in yeast two-hybrid assays.  AHPs act as positive regulators of MSP-mediated CK signaling.  AHP2 interacts with ARR2,10 in yeast two-hybrid assays.  Analogous with two-component systems in other organisms. | [13,54]⁠  [78]  [55]⁠  [55]⁠ |
| ARF –| A-ARRs 15,7 | The *ARR7* and *ARR15* transcripts are reduced in inflorescence stems treated with auxin.  *ARR7* and *ARR15* have increased levels of expression in *yucca* mutants, which lack endogenous auxin.  ARR7 and ARR15 levels are strongly elevated in apices of the arf5/monopteros mutant.  *ARR7* and *ARR15* are regulated by auxin and by the CK signaling pathway – independently, and on different regions of their promoters.  *Note: In the embryo and in the root, this interaction has the opposite sign: auxin positively regulates *ARR7* and *ARR15* and therefore downregulates CK. | [56]⁠  [18,57]⁠  [56]⁠  [56]⁠  [57]⁠ |
| ARF → HB8 | Expression of PIN1:GFP and DR5rev::GFP precede Athb8::GUS expression in developing leaves.  *HB8* is responsive to auxin. It has a functional auxin response sequence that, when mutated, eliminates *HB8* response to auxin.  *HB8* and *MP* expression patterns in the leaves overlap.  Interaction proven by co-precipitation. | [58]⁠  [58]⁠  [59]⁠  [58]⁠ |
| ARF → AHP6 | Microarray data show that *AHP6* is downregulated in *mp* mutant seedlings.  A 10-fold increase in *AHP6* expression in wild-type roots is observed after 2 hr incubation with IAA. | [60]⁠  [18]⁠ |
| ASL → VND | The generation of tracheary-like cells by *ASL19/20* overexpression required *VND6* and *VND7* activities and *ASL20* overexpression induced ectopic expression of *VND7*.  *VND6* and *VND7* mRNA levels were reduced in dwarfed plants with an *ASL* loss-of-function mutation. | [6,32]⁠  [61]⁠ |
| BR → HB8 | In *Zinnia elegans*, inhibitors of brassinosteroid biosynthesis completely suppress the accumulation of transcripts for ZeHB-10, -11, and -12 (HD-Zip III homeobox genes similar in sequence and function to HB8). Also, BR immediately induces the accumulation of these transcripts in developing xylem cells. Also, in Zinnia mesophyll cell cultures, BRs have been shown to regulate xylem differentiation.  In Arabidopsis, BR receptors are predominantly expressed in provascular tissues. Double- and triple-mutants of BR receptors suggest a function in the elaboration of xylem. Mutants with reduced BRI1 receptor activity, signaling, or levels have fewer vascular bundles, while mutants with increased BR levels have an increased number of vascular bundles. | [1–4]⁠  [5,6]⁠ |
| CK→ A-ARRs | Exogenous CKs promptly uppregulate several A-ARRs in the run-on assay.  AHK2,3,4, which indirectly regulate B-ARRs, enhance the promoter of *ARR6*.  There are multiple ARR2 binding motifs, (G/A)GAT(T/C), in the promoter regions of *ARR6* and other cytokinin-inducible genes. | [17]  [8]⁠  [7]⁠ |
| CKs (via ARRs)–| AHP6 | Predicted interaction (in the shoot; could be direct or indirect).  Informed by the following data:  Expression domain of AHP6 in *cre1 ahk3* roots is slightly broader than in wild-type. In roots of both *wol* and *cre1 ahk2 ahk3*, the *AHP6* expression pattern is expanded. Also in the root, the *AHP6* transcript is downregulated after a treatment with CKs, and the level of fluorescence in AHP6prom::GFP is also reduced by exogenous CKs. | [9]⁠ |
| CK (via ARRs) → CKX | AtCKX promoter-GUS marker is increased by CKs  within the gene-specific domains (e.g. AtCKX5::GUS in the root meristem).  In *Zea mays*, CKX are locally induced by synthetic and natural CKs. Also in *Zea mays*, the expression patterns of CKX correlate with CK levels. | [10]⁠  [11,12]⁠ |
| CK → AHKs  (as part of the MSP) | AHKs sequence contains a receiver domain.  AHK2 complements yeast mutants in a cytokinin-dependent manner.  AHK3 complements yeast mutants in a cytokinin-dependent manner.  AHK4 (WOL/CRE1) mutant showed a lack of cytokinin responses.  AHK4 complemented mutants in yeast and E. coli.  AHK4 binds active CKs in yeast assays.  Plant carrying different combinations of AHK2, AHK3 and AHK4 mutant alleles show CK deficiency phenotypes.  *Note: AHK3 and AHK4 differ in ligand specificity. | [13,17]⁠  [13]⁠  [13,15,16]⁠    [14]⁠  [13]⁠  [15,16]⁠  [78,79] |
| CK → PIN7 radial localization | Predicted interaction (could be direct or indirect)  Informed by the following data:  During the specification of root vascular cells in *Arabidopsis thaliana*, CK regulates the radial localization of PIN7.  Expression of PIN7:GFP and PIN7::GUS is upregulated by CK with no significant influence of ethylene.  In the root, CK signaling is required for the CK regulation of *PIN1*, *PIN3*, and *PIN7*. Their expression is altered in wol, cre1, ahk3 and ahp6 mutants. | [18]⁠  [18,20]⁠  [19]⁠ |
| CK→ APL | Predicted interaction (could be direct or indirect)  Consistent with the fact that *APL* overexpression prevents or delays xylem cell differentiation, as does CKs.  Partially supported by microarray data and phloem-specific expression patterns of CK response factors. | [21]  (TAIR, ExpressionSet:1005823559, [22]⁠) |
| CKX –| CK | Ectopic overexpression of CKXs results into decreased endogenous CK levels and decrease in the sensitivity to exogenous CKs.  CKXs (1-7) catalyze the irreversible degradation of CKs.  Double mutants including the *ckx3-1* allele formed more ﬂowers, indicating a more active inﬂorescence meristem.  *ckx3 ckx5* mutants formed a stem with a diameter 15% larger than the WT caused by an increased cambial activity. | [19,24]  [23]⁠    [24]⁠  [23]⁠ |
| HB8 –| KAN | Predicted interaction (could be direct or indirect)  Informed by the following data:  Antagonistic interaction is inferred from the observation that gain-of-function mutants of other HD-ZIPIII members and triple k*an1 kan2 kan3* loss-of-function mutants have adaxialized lateral organs and vascular bundles in which xylem surrounds phloem. Furthermore, these genes have complementary expression patterns. | (Reviewed in [25]⁠). |
| HB8 → BR | Predicted interaction (could be direct or indirect)  Consistent with the fact that procambial cells in which HB8 is expressed produce BR in the presence of IAA and CK. | [26]⁠ |
| IAA (via ARF) –| IPT | In Arabidopsis seedlings treated with NAA, pool sizes of several of the major cytokinin intermediates and end products are rapidly and significantly reduced.  The biosynthetic rate of ZMP was significantly reduced after auxin treatment. Correspondingly, treated plants show reduced levels of Z type cytokinins, whereas the levels of iP-type cytokinins are less affected.  PsIPT2:GUS in transgenic *Arabidopsis*, was repressed by an IAA.  *Note: In the root, IAA seems to upregulate IPT5, both directly and via the downregulation of SHY2. | [30]⁠  [28]⁠  [29]⁠  [28]⁠ |
| IAA → ASL | Levels of *ASL20* mRNA, but not of *ASL19* mRNA, increased within 30 min after treatment with 1-N-naphthylphthalamic acid (NPA), an inhibitor of polar auxin transport, and this increase was dependent on auxin signaling via *ARF7*. This results suggest that auxin enhances the expression of *ASL20* in tissue-specific way  *ASL* is a xylem marker and, in *Zinnia*, single isolated mesophyll cells transdifferentiate into xylem tracheary cells within 3 d when cultured in the presence of auxin and cytokinin. | [31]⁠  [6,32]⁠  [33]⁠ |
| IAA → CKXs | The *Arabidopsis* microarray gene expression database Genevestigator showed IAA-activated expression of *AtCKX1* and *AtCKX6* (2.9- and 7.9-fold up-regulation, respectively).  Breakdown of radiolabelled cytokinin is enhanced in response to 1-naphthylacetic acid (NAA).  Note: Semiquantitative RT-PCR showed, however, that NAA causes only subtle changes in abundance of *AtCKX* transcripts, and that sometimes these changes are towards negative regulation. | [10]⁠  [reviewed in 10]⁠  [10] |
| IAA →PXY | *PXY* is required for a stable auxin-dependent increase in *WOX4* mRNA. | [34]⁠ |
| IPT → CK | Overexpression of *AtIPT4* or *AtIPT8* confers cytokinin-independent shoot formation on calli, and overexpression of *AtIPT1*, 3, 4, 5, 7, or 8 causes increased iP-type cytokinin levels *in planta*.  The rate-limiting step of CK biosynthesis is catalyzed by enzymes encoded by the *IPT* gene family. | [10,37]⁠  [35,36]⁠ |
| KAN1 → MIR165/66 | Predicted interaction (could be direct or indirect)  Informed by the following data:  Expression patterns of *KAN1* and its inhibitory effect on HD ZIP III genes, which are in turn regulated by MIR165-6 | (Reviewed in [38]⁠) |
| MiR165/166 –| HB8 | In *pATHB8::miR165* plants, the expression of all the HD-ZIP III genes is reduced in procambium, and procambial cells fail to differentiate into xylem cells, but proliferate actively to produce many more procambium cells.  MIR165 and 66 have sequences that are complementary to the START domain of HD-ZIP III genes.  Mature forms of miR165 and miR166, non-cell-autonomously suppress HD-ZIP III transcripts in the root peripheral stele.  Expression of HD-ZIP III genes is suppressed by the action of miR165 and miR166.  The root radial pattern of a dominant *phb-1d* mutant that expresses miRNA-resistant PHB (HD-ZIP III) transcripts is altered, having fewer stele cell files. | [41]⁠  [39]⁠  [5,6,40]⁠  [42]⁠  [42] |
| TDIF (CLE41/44) –| TDR/PXY  (transcriptionally) | In *35S::CLE41* plants, there is a reduction in *PXY* expression in the inflorescence stem and hypocotyl.  *PXY* mRNA is elevated in *pxy* mutants. | [44]⁠  [43]⁠ |
| TDIF → TDR/PXY  (ligand-mediated) | Small peptides of the CLE family (CLE41, 44 and CLE42) act as tracheary differentiation inhibitors in both *Zinnia* and *Arabidopsis*.  TDIF binds *in vitro* specifically to the TDR(PXY) receptor, whose expression is restricted to procambium. This interaction is not observed between TDR and other CLE peptides.  Plants homozygous for *tdr* are insensitive to TDIF. | [43,45–47]⁠  [45]⁠  [45]⁠ |
| TDR/PXY → WOX4 | *WOX4* transcript is upregulated rapidly after the application of TDIF in a TDR-dependent and specific manner.  This rapid activation of *WOX4* expression is not observed in the *tdr-1* mutant (also called *pxy-5*). | [48,49]⁠  [48]⁠ |
| TDR(PXY) –| APL | Addition of CLE6 and 41 caused a loss of *APL* expression in the hypocotyl. | [47]⁠ |
| VND → ASL | The mRNA levels of *ASL18* and *19* increased in inducible *VND6* and *VND7* overexpression mutants.  GUS staining assays showed ectopic expression of both ASL promoter–reporter genes in the nonvascular tissues of plants overexpressing *VND7* and *VND6*.  Overexpression of *ASL19* or *ASL20* induced transdifferentiation of cells from nonvascular tissues into TE-like cells, similar to those formed upon *VND6* and *VND7* overexpression.  *Pro**VND7:EGFP-GUS* was expressed in more non-lignified tracheary elements than *ASL19* and *20*, suggesting that, in differentiating TEs, *VND7* expression occurs prior to that of *ASL19* and *ASL20*. | [6,32]⁠  [32]  [32]  [32] |
| WOX4 → HB8 | Down-regulated *WOX4* expression by RNAi exhibit reduced vascular development and overaccumulate undifferentiated ground tissue. Moreover, the expression of *AtHB8* is reduced and delayed in young primordia.  TDIF, which upregulates *WOX4* via TDR, promoted the expression of the procambium-specific marker gene, *HB8*. | [49]⁠  [45]⁠ |

**Table 2. Table summarizing the reported expression or presence patterns of the network** elements.

| **Element** | **Expression, synthesis or presence in the vascular tissues of *Arabidopsis thaliana*** | | | | **Mobility** |
| --- | --- | --- | --- | --- | --- |
|  | **Xylem** | **Procambium** | **Phloem** | **Shoot/root distribution** |  |
| AHP6 | In the root, it has a bisymmetric  maximum in the xylem axis [9,18].⁠ |  |  | *AHP6* is also expressed in the shoot apex and  young leaves [9].⁠ |  |
| APL |  |  | Phloem marker  [21]⁠ | Expressed in both roots and shoots [21].⁠ |  |
| A-ARRs |  | *ARR5* is expressed in the root vasculature [9,18]⁠ and more precisely in the root procambial cells in the proximal meristem [17].⁠ |  | *ARR15* is expressed in the root vasculature [27]⁠  *ARR3,4,5,9* are expressed in the root vasculature [70]⁠ |  |
| B-ARRs |  | *ARR1* appears to be expressed in the root procambium [70]⁠ |  | *ARR10* and *12* are expressed in the root vasculature [76]⁠ |  |
| ASL19, 20 | *ASL19* and *ASL20* are expressed in immature tracheary elements [32]⁠ |  |  | Detected in the vascular  tissues of leaves, hypocotyls, roots, developing floral organs, and siliques. [32]⁠ |  |
| BRL1 |  | In inﬂorescence stems, *BRL1* expression is associated with the procambial cells of the vascular bundles [71]⁠ |  |  |  |
| CK | Mostly (tZ)-type CK [65,66]⁠ | In a model for the root, CK are mostly concentrated in the PC [18]⁠ | Mostly (iP)-type cytokinins in phloem sap  [65,66]⁠  CK moving rootward through the phloem are necessary for root vascular pattern formation [73]⁠ | Radio labeled BA shows a peak in the root tip and in the transition zone, where the vascular tissue is differentiated. Marked CK are shown to move basipetally (rootwards) mainly through the phloem [73]⁠.  tZ-type cytokinins seem to be used  as an acropetal messenger and iP-type cytokinins as a basipetal one (rev. in [66]⁠) | When plasmodesmatal blockage is  induced in the root, transport and unloading  of the CK BA is severely affected and its concentration diminished [73]⁠. |
| CKX |  | Confined to proliferating cells of young tissues, like the shoot apex (AtCKX1, CKX2), young leaves (AtCKX4, CKX5), and procambial region of the root meristem (AtCKX5) [10]⁠. | *CKX6* expressed in the phloem of leaf vasculature and root  [10,72]⁠. | *IPT* and *CKX* partially share expression in meristematic tissues, which further suggest a locally restricted CK action [10]⁠. |  |
| HB8 | [5] found in xylem precursors both in Zinnia and Arabidopsis. | *HB8* is a marker of preprocambial cells in the shoot [58,67,68]⁠. |  |  |  |
| KANADI genes (KAN1-4) |  |  | Different genes of this family are expressed in the phloem of leaves and embryo [40]. |  |  |
| IAA | DR5::GUS levels peak in the xylem at the base of the inflorescence stem, specifically at the vascular bundles, having a periodic expression in the procambial and differentiating xylem cells [3]⁠.  In the root, DR5::GUS revealed that the GUS reporter peaked in the protoxylem strands and was absent from the adjacent pericycle [18]⁠  In the root, it has a bisymmetric  maximum in the xylem axis [62]⁠ | DR5::GUS levels peak in the procambium at the base of the inflorescence stem, specifically at the vascular bundles, having a periodic expression in the procambial and differentiating xylem cells [5]⁠.  In the root, DR5::GUS revealed that the GUS reporter peaked in the protoxylem strands and was absent from the adjacent pericycle [19]  In the root, it has a bisymmetric  maximum in the xylem axis [62]⁠. | DR5revpro:GFP is active at two different positions along the inflorescence stem (10 millimeters above the uppermost rosette leaf and at the position of the uppermost rosette leaf) has activity mainly in the phloem cells and in single cortex cells in interfascicular regions [34]⁠. | The shoot apex represents a major source of auxin, from which it is  transported basipetally, but there are local (sometimes transient) maxima in other parts of the plant [80].  The bulk basipetal transport of 14C-labeled IAA was impaired when phloem transport was blocked, but still the auxin pattern in the RAM was formed [18]⁠ | Long-range transport via the phloem, mostly from source tissues (young leaves and floral buds) to root and shoot tips, and short-range polar auxin transport [73]⁠  When plasmodesmatal blockage is  induced in the root, transport and unloading  of IAA is severely affected and its concentration diminished [77]⁠. |
| IPT |  | Soon after germination, its activity  is localized  in the root procambium [69]. | Strong activity in the root phloem [69]⁠. |  | IPT and CKX partially share expression in merisitematic tissues, which further suggest paracrine CK action [10]⁠. |
| MP (ARF) |  |  |  | Expressed in leaf veins, roots, stem, SAM, flower, embryo [56,74,75]⁠. |  |
| MIR165-66  (micro RNA) |  |  |  | Mobile between cells.  In the root, MIR165/166 genes are expressed  specifically in the endodermis in a SHR- and SCR-dependent manner [41]. |  |
| PIN | In the shoot, it has been shown that PIN1 is expressed in the procambium and xylem cells, at the basal side and in a fraction of the lateral cell membranes [3,63]⁠. | In the shoot, it has been shown that PIN1 is expressed in the procambium and xylem cells, at the basal side and in a fraction of the lateral cell membranes[3,59,63]⁠  In the root, PIN7 is highly expressed in procambial cells and seems to transport IAA from these cells to protoxylem cells [18]⁠ |  |  |  |
| TDIF |  |  | Expressed specifically in the phloem of the shoot (CLE41,44) [45,48]⁠. |  | This small peptide moves towards procambial cells [43,45,48]⁠. |
| TDR/PXY |  | Expressed in the procambial cells of the shoot [45,48]⁠. |  | Although expressed in both shoot and root, *pxy* roots do not exhibit obvious phenotypic alterations [44], suggesting that it has a predominating role in the development of the shoot vasculature. |  |
| VND6 and VND7 | Regulators initiating metaxylem and protoxylem vessel differentiation in the root and shoot  RELATED NAC-DOMAIN PROTEIN6 (VND6) and VND7 [32,61,64]⁠ |  |  |  |  |
| WOX4 |  | Expressed specifically in the shoot procambial and cambial cells [34,45,48,49]⁠. |  |  |  |

**Table 3. Logical rules defining the model dynamics.** These rules determine the state (0,1) or (0,1,2) of each of the nodes depending on the state of its regulators (Figure 1). The rules are grounded on experimental evidence (Table 1 and Table 2) and expressed by means of the standard Boolean operators (and, or). * Indicate newly postulated interactions.

| **Network node** | **Dynamical rule** |
| --- | --- |
| CK | 2 If ipt=1 and ckx=0  1 If ipt=1 and ckx=1  0 else |
| CKX | 1 If barr>0 or arf=2  0 else |
| AHKs | ahk=ck |
| AHPs | 2 If ahk=2 and ahp6=0 and aarr=0  1 If ahk=2 and (ahp6+aarr<2)  1 If ahk=1 and ahp6<1  0 else |
| B-Type ARRs | 1 If ahp>0  0 else |
| A-Type ARRs | 1 If arf<2 and ahp>0  0 else |
| WOX4 | wox4=apxy |
| PXY | 1 If tdif<2 and arf>0  0 else |
| Active PXY  (APXY) | 1 If pxy=1 and tdif>0  0 else |
| TDIF | tdif=tdif |
| ARF | arf=iaa |
| AHP6* | 1 If arf=2 or (barr=0 and arf=1)  0 else |
| IPT | 1 If barr=1 and arf<2  0 else |
| IAA | iaa=iaa |
| PIN localization | pin_radial_localization=barr |
| HB8 | 1 If arf=2 or (mir<2 and (wox4+arf+br>1))  0 else |
| MIR165/6* | mir=kan |
| KAN* | 1 if hb8=0  0 else |
| APL1* | 1 If apxy=0 and barr>0  0 else |
| VND | vnd=asl |
| ASL | 1 If arf=2 or (arf=1 and vnd=1)  0 else |
| BR* | 1 If hb8=1  0 else |

**Table 4. Analysis of model robustness to changes in the given parameter values.** Taking Diaa=0.3; Dtdif=0.25; Dmir=0.25; Degmir=0.1; Degiaa=0.5; Degtdif=0.1 as reference, and varying every parameter, one by ene, from 0 to 0.5, with an increment of h=0.01, the output of each simulation is compared with the reference simulation. The most sensitive parameters are those of TDIF, probably because additional regulatory inputs acting on TDIF are still lacking. Changes in the MIR parameters do not affect the overall patterns or the state of other nodes, but only affect the formation of a MIR gradient that might be important for the specification of subtypes of vascular cells.

| **Parameter** | **IAA** | **MIR165/6** | **TDIF** |
| --- | --- | --- | --- |
| Mobility (transport for IAA; diffusion for MIR and TDIF) | 0.26-0.5  (< 0.26: no xylem) | 0.11-0.39  (only affecting gradient of MIR) | 0.21-0.3  (< 0.21: no procambial genes APXY and WOX4; > 0.3: expression of procambial genes outside procambium) |
| Degradation | 0.3-0.5  (< 0.3: CKs are lost in procambium) | 0 - 0.24  (only affecting gradient of MIR) | 0 – 0.19  (> 0.19: expression of procambial genes outside procambium) |


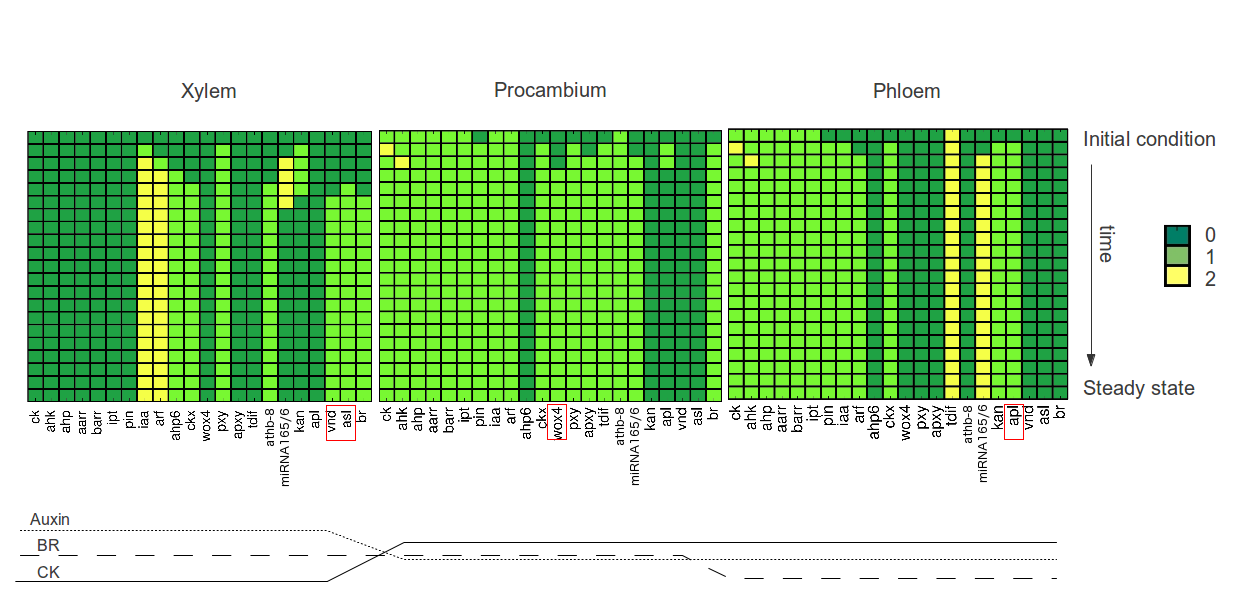


**Supplementary Figure 1. Simulation of the modeling time course.** Each of the three modeled compartments contains the same molecular regulatory network. The rows correspond to the activation profile of each network element; dark green stands for 0, light green for 1, and yellow for 2 (see color code in the figure). The first row (top) corresponds to the initial conditions (see the Results for details) and each of the rows below to activation profiles in subsequent time steps. The last row (bottom) corresponds to the final steady state in which the specific cell-type activation profiles are sustained, given the dynamical rules and cell-to-cell processes considered in the model. Below, the steady activation profiles for the hormones IAA, CK and BR are detailed.

**Supplementary References**

1. Li J, Chory J (1997) A putative leucine-rich repeat receptor kinase involved in brassinosteroid signal transduction. Cell 90: 929–938.

2. Wang ZY, Nakano T, Gendron J, He J, Chen M, et al. (2002) Nuclear-localized BZR1 mediates brassinosteroid-induced growth and feedback suppression of brassinosteroid biosynthesis. Dev Cell 2: 505–513.

3. Ibañes M, Fàbregas N, Chory J, Caño-Delgado AI (2009) Brassinosteroid signaling and auxin transport are required to establish the periodic pattern of Arabidopsis shoot vascular bundles. Proc Nat Acad Sci USA 106: 13630–13635.

4. Szekeres M, Németh K, Koncz-Kálmán Z, Mathur J, Kauschmann a, et al. (1996) Brassinosteroids rescue the deficiency of CYP90, a cytochrome P450, controlling cell elongation and de-etiolation in Arabidopsis. Cell 85: 171–182.

5. Ohashi-Ito K, Fukuda H (2003) HD-zip III homeobox genes that include a novel member, ZeHB-13 (Zinnia)/ATHB-15 (Arabidopsis), are involved in procambium and xylem cell differentiation. Plant Cell Physiol 44: 1350–1358.

6. Ohashi-Ito K, Fukuda H (2010) Transcriptional regulation of vascular cell fates. Curr Op Plant Biol 13: 670–676.

7. Kondo Y, Hirakawa Y, Kieber JJ, Fukuda H (2011) CLE peptides can negatively regulate protoxylem vessel formation via cytokinin signaling. Plant Cell Physiol 52: 37–48.

8. Sakai H, Honma T, Aoyama T, Sato S, Kato T, et al. (2001) ARR1, a transcription factor for genes immediately responsive to cytokinins. Science 294: 1519–1521.

9. Mahönen AP, Bishopp A, Higuchi M, Nieminen KM, Kinoshita K, et al. (2006) Cytokinin signaling and its inhibitor AHP6 regulate cell fate during vascular development. Science 311: 94–98.

10. Werner T, Köllmer I, Bartrina I, Holst K, Schmülling T (2006) New insights into the biology of cytokinin degradation. Plant Biol 8: 371–381.

11. Brugiere N, Jiao S, Hantke S, Zinselmeier C, Roessler JA, et al. (2003) Cytokinin Oxidase Gene Expression in Maize Is Localized to the Vasculature , and Is Induced by Cytokinins , Abscisic Acid , and Abiotic Stress. Plant Physiol 132: 1228–1240.

12. Schmülling T, Werner T, Riefler M, Krupková E, Bartrina et al. (2003) Structure and function of cytokinin oxidase/dehydrogenase genes of maize, rice, Arabidopsis and other species. J Plant Res 116: 241–252.

13. Hwang I, Sheen J (2001) Two-component circuitry in Arabidopsis cytokinin signal transduction. Nature 413: 383–389.

1. Suzuki T, Sakurai K, Ueguchi C, Mizuno T (2001) Two types of putative nuclear factors that physically interact with histidine-containing phosphotransfer (Hpt) domains, signaling mediators in His-to-Asp phosphorelay, in Arabidopsis thaliana. Plant Cell Physiol 42: 37–45.
2. Hwang I, Chen H, Sheen J (2002) Two-Component Signal Transduction Pathways in Arabidopsis. Plant Physiol 129: 500–515.

16. Sheen J (2002) Phosphorelay and transcription control in cytokinin signal transduction. Science 296: 1650–1652.

17. Agostino IBD, Derue J, Kieber JJ, Carolina N, Hill C, et al. (2000) Characterization of the Response of the Arabidopsis Response Regulator Gene Family to Cytokinin 1. Plant Physiol 124: 1706–1717.

18. Bishopp A, Help H, El-Showk S, Weijers D, Scheres B, et al. (2011) A mutually inhibitory interaction between auxin and cytokinin specifies vascular pattern in roots. Curr Biol 21: 917–926.

19. Pernisová M, Klíma P, Horák J, Válková M, Malbeck J, et al. (2009) Cytokinins modulate auxin-induced organogenesis in plants via regulation of the auxin efflux. Proc the Nat Acad Sci USA 106: 3609–3614.

20. Ruzicka K, Simásková M, Duclercq J, Petrásek J, Zazímalová E, et al. (2009) Cytokinin regulates root meristem activity via modulation of the polar auxin transport. Proc the Nat Acad Sci USA 106: 4284–4289.

21. Bonke M, Thitamadee S, Mähönen AP, Hauser M, Helariutta Y (2003) APL regulates vascular tissue identity in Arabidopsis. Nature 426: 181–186.

22. Zwack PJ, Shi X, Robinson BR, Gupta S, Compton MA, et al. (2012) Vascular Expression and C-terminal Sequence Divergence of Cytokinin Response Factors in Flowering Plants. Plant Cell Physiol 53: 1683-95.

23. Bartrina I, Otto E, Strnad M, Werner T, Schmülling T (2011) Cytokinin regulates the activity of reproductive meristems, flower organ size, ovule formation, and thus seed yield in Arabidopsis thaliana. Plant Cell 23: 69–80.

24. Werner T, Motyka V, Laucou V, Smets R, Onckelen HV (2003) Cytokinin-Deficient Transgenic Arabidopsis Plants Show multiple developmental alterations indicating opposite functions of cytokinins in the regulation of Shoot and Root Meristem Activity. Plant Cell 15: 2532–2550.

25. Dettmer J, Elo A, Helariutta Y (2009) Hormone interactions during vascular development. Plant Mol Biol 69: 347–360.

26. Yamamoto R, Fujioka S, Demura T, Takatsuto S, Yoshida S, et al. (2001) Brassinosteroid levels increase drastically prior to morphogenesis of tracheary elements. Plant Physiol 125: 556–563.

27. To JPC, Haberer G, Ferreira FJ, Derue J, Schaller GE, et al. (2004) Type-A Arabidopsis Response Regulators Are Partially Redundant Negative Regulators of Cytokinin Signaling. Plant Cell 16: 658–671.

28. Nordström A, Tarkowski P, Tarkowska D, Norbaek R, Astot C, et al. (2004) Auxin regulation of cytokinin biosynthesis in Arabidopsis thaliana: a factor of potential importance for auxin-cytokinin-regulated development. Proc the Nat Acad Sci USA 101: 8039–8044.

29. Tanaka M, Takei K, Kojima M, Sakakibara H, Mori H (2006) Auxin controls local cytokinin biosynthesis in the nodal stem in apical dominance. Plant J 45: 1028–1036.

30. Dello Ioio R, Nakamura K, Moubayidin L, Perilli S, Taniguchi M, et al. (2008) A genetic framework for the control of cell division and differentiation in the root meristem. Science 322: 1380–1384.

31. Fukuda H, Komamine A (1980) Establishment of an Experimental System for the Study of Tracheary Element Differentiation from Single Cells Isolated from the Mesophyll of Zinnia elegans. Plant Physiol 65: 57–60.

32. Soyano T, Thitamadee S, Machida Y, Chua N-H (2008) ASYMMETRIC LEAVES2-LIKE19/LATERAL ORGAN BOUNDARIES DOMAIN30 and ASL20/LBD18 regulate tracheary element differentiation in Arabidopsis. Plant Cell 20: 3359–3373.

33. Fukuda H (2004) Signals that control plant vascular cell differentiation. Nat Rev Mol Cell Biol 5: 379–391.

34. Suer S, Agusti J, Sanchez P, Schwarz M, Greb T (2011) WOX4 Imparts Auxin Responsiveness to Cambium Cells in Arabidopsis. Plant Cell 23: 3247–3259.

35. Sakakibara H (2006) Cytokinins: activity, biosynthesis, and translocation. Ann Rev Plant Biol 57: 431–449.

36. Sun J, Niu Q, Tarkowski P, Zheng B, Tarkowska D (2003) The Arabidopsis AtIPT8 / PGA22 Gene Encodes an Isopentenyl Transferase That Is Involved in De Novo Cytokinin Biosynthesis. Plant Physiol 131: 167–176.

37. Kamada-Nobusada T, Sakakibara H (2009) Molecular basis for cytokinin biosynthesis. Phytochemistry 70: 444–449.

38. Caño-Delgado A, Lee J-Y, Demura T (2010) Regulatory mechanisms for specification and patterning of plant vascular tissues. Ann Rev Cell Dev Biol 26: 605–637.

39. Sakaguchi J, Watanabe Y (2012) miR165/166 and the development of land plants. Dev Growth Diff 54: 93–99.

40. Ilegems M, Douet V, Meylan-Bettex M, Uyttewaal M, Brand L, et al. (2010) Interplay of auxin, KANADI and Class III HD-ZIP transcription factors in vascular tissue formation. Development 137: 975–984.

41. Carlsbecker A, Lee J-Y, Roberts CJ, Dettmer J, Lehesranta S, et al. (2010) Cell signalling by microRNA165/6 directs gene dose-dependent root cell fate. Nature 465: 316–321.

42. Miyashima S, Koi S, Hashimoto T, Nakajima K (2011) Non-cell-autonomous microRNA165 acts in a dose-dependent manner to regulate multiple differentiation status in the Arabidopsis root. Development 138: 2303–2313.

43. Etchells JP, Turner SR (2010) The PXY-CLE41 receptor ligand pair defines a multifunctional pathway that controls the rate and orientation of vascular cell division. Development 137: 767–774.

44. Fisher K, Turner S (2007) PXY, a receptor-like kinase essential for maintaining polarity during plant vascular-tissue development. Curr Biol17: 1061–1066.

45. Hirakawa Y, Shinohara H, Kondo Y, Inoue A, Nakanomyo I, et al. (2008) Non-cell-autonomous control of vascular stem cell fate by a CLE peptide/receptor system. Proc Nat Acad Sci USA 105: 15208–15213.

46. Ito Y, Nakanomyo I, Motose H, Iwamoto K, Sawa S, et al. (2006) Dodeca-CLE peptides as suppressors of plant stem cell differentiation. Science 313: 842–845.

47. Whitford R, Fernandez A, De Groodt R, Ortega E, Hilson P (2008) Plant CLE peptides from two distinct functional classes synergistically induce division of vascular cells. Proc Nat Acad Sci USA 105: 18625–18630.

48. Hirakawa Y, Kondo Y, Fukuda H (2010) TDIF peptide signaling regulates vascular stem cell proliferation via the WOX4 homeobox gene in Arabidopsis. Plant Cell 22: 2618–2629.

49. Ji J, Shimizu R, Sinha N, Scanlon MJ (2010) Analyses of WOX4 transgenics provide further evidence for the evolution of the WOX gene family during the regulation of diverse stem cell functions. Plant Signal Behav 5: 916–920.

50. Brandstatter I, Kieber JJ (1998) Two genes with similarity to bacterial response regulators are rapidly and specifically induced by cytokinin in Arabidopsis. Plant Cell 10: 1009–1019.

51. Taniguchi M, Kiba T, Sakakibara H, Ueguchi C, Mizuno T, et al. (1998) Expression of Arabidopsis response regulator homologs is induced by cytokinins and nitrate. FEBS lett 429: 259–262.

52. Kiba T, Taniguchi M, Imamura a, Ueguchi C, Mizuno T, et al. (1999) Differential expression of genes for response regulators in response to cytokinins and nitrate in Arabidopsis thaliana. Plant Cell Physiol 40: 767–771.

53. Punwani JA, Kieber JJ (2010) [Localization of the Arabidopsis histidine phosphotransfer proteins](http://www.ncbi.nlm.nih.gov/pubmed/20484973) is independent of cytokinin. Plant Cell Physiol 5: 896–898.

54. Chang C, Stewart RC (1998) Update on Signal Transduction The Two-Component System 1 Regulation of Diverse Signaling Pathways in Prokaryotes and Eukaryotes. Plant Mol Biol: 723–731.

55. Suzuki T, Imamura A, Ueguchi C, Mizuno T (1998) Histidine-containing phosphotransfer (HPt) signal transducers implicated in His-to-Asp phosphorelay in Arabidopsis. Plant Cell Physiol 39: 1258–1268.

56. Zhao Z, Andersen SU, Ljung K, Dolezal K, Miotk A, et al. (2010) Hormonal control of the shoot stem-cell niche. Nature 465: 1089–1092.

57. Müller B, Sheen J (2008) Cytokinin and auxin interaction in root stem-cell specification during early embryogenesis. Nature 453: 1094–1097.

58. Donner TJ, Sherr I, Scarpella E (2009) Regulation of preprocambial cell state acquisition by auxin signaling in Arabidopsis leaves. Development 136: 3235–3246.

59. Scarpella E, Marcos D, Berleth T (2006) Control of leaf vascular patterning by polar auxin transport. Genes Dev 20:1015–1027.

60. Schlereth A, Möller B, Liu W, Kientz M, Flipse J, et al. (2010) MONOPTEROS controls embryonic root initiation by regulating a mobile transcription factor. Nature 464: 913–916.

61. Kubo M, Udagawa M, Nishikubo N, Horiguchi G, Yamaguchi M, et al. (2005) Transcription switches for protoxylem and metaxylem vessel formation. Genes Dev 19: 1855–1860.

62. De Smet I, Tetsumura T, De Rybel B, Frey NFD, Laplaze L, et al. (2007) Auxin-dependent regulation of lateral root positioning in the basal meristem of Arabidopsis. Development 134: 681–690.

63. Gälweiler L, Wisman E, Mendgen K, Yephremov A, Palme K (2011) Regulation of Polar Auxin Transport by AtPIN1 in Arabidopsis Vascular Tissue. Science 283:2226-30.

64. Yamaguchi M, Kubo M, Fukuda H, Demura T (2008) Vascular-related NAC-DOMAIN7 is involved in the differentiation of all types of xylem vessels in Arabidopsis roots and shoots. Plant J 55: 652–664.

65. Hirose N, Takei K, Kuroha T, Kamada-Nobusada T, Hayashi H, et al. (2008) Regulation of cytokinin biosynthesis, compartmentalization and translocation. J Exp Bot 59: 75–83.

66. Kudo T, Kiba T, Sakakibara H (2010) Metabolism and long-distance translocation of cytokinins. J Int Plant Biol 52: 53–60.

67. Baima S, Possenti M, Matteucci a, Wisman E, Altamura MM, et al. (2001) The arabidopsis ATHB-8 HD-zip protein acts as a differentiation-promoting transcription factor of the vascular meristems. Plant Physiol 126: 643–655.

68. Baima S, Nobili F, Sessa G, Lucchetti S, Ruberti I, et al. (1995) The expression of the Athb-8 homeobox gene is restricted to provascular cells in Arabidopsis thaliana. Development 121: 4171–4182.

69. Miyawaki K, Matsumoto-Kitano M, Kakimoto T (2004) Expression of cytokinin biosynthetic isopentenyltransferase genes in Arabidopsis : tissue specificity and regulation by auxin, cytokinin, and nitrate. Plant J 37: 128–138.

70. Kiba T, Yamada H, Sato S, Kato T, Tabata S, et al. (2003) The type-A response regulator, ARR15, acts as a negative regulator in the cytokinin-mediated signal transduction in Arabidopsis thaliana. Plant Cell Physiol 44: 868–874.

71. Caño-Delgado A, Yin Y, Yu C, Vafeados D, Mora-García S, et al. (2004) BRL1 and BRL3 are novel brassinosteroid receptors that function in vascular differentiation in Arabidopsis. Development 131: 5341–5351.

72. Werner T, Schmülling T (2009) Cytokinin action in plant development. Curr Op Plant Biol 12: 527–538.

73. Bishopp A, Lehesranta S, Vatén A, Help H, El-Showk S, et al. (2011) Phloem-transported cytokinin regulates polar auxin transport and maintains vascular pattern in the root meristem. Curr Biol 21: 927–932.

74. Donner TJ, Sherr I, Scarpella E (2010) Auxin signal transduction in Arabidopsis vein formation. Development 5: 70–72.

75. Gordon SP, Chickarmane VS, Ohno C, Meyerowitz EM (2009) Multiple feedback loops through cytokinin signaling control stem cell number within the Arabidopsis shoot meristem. Proc Nat Acad Sci USA 106: 16529–16534.

76. Yokoyama A, Yamashino T, Amano Y-I, Tajima Y, Imamura A, et al. (2007) Type-B ARR transcription factors, ARR10 and ARR12, are implicated in cytokinin-mediated regulation of protoxylem differentiation in roots of Arabidopsis thaliana. Plant Cell Physiol 48: 84–96.

1. Robert HS, Friml J (2009) Auxin and other signals on the move in plants. Nature Chem Biol 5: 325–332.
2. Hutchison CE, Li J, Argueso C, Gonzalez M, Lee E, et al. (2006) The Arabidopsis histidine phosphotransfer proteins are redundant positive regulators of cytokinin signaling. Plant Cell 18: 3073-87.
3. Nishimura C, Ohashi Y, Sato S, Kato T, Tabata S, et al. (2004) Histidine kinase homologs that act as cytokinin receptors possess overlapping functions in the regulation of shoot and root growth in Arabidopsis. Plant Cell 16: 1365-77.
4. Benková E, Michniewicz M, Sauer M, Teichmann T, Seifertová D, et al. (2003) Local, efflux-dependent auxin gradients as a common module for plant organ formation. Cell 115: 591-602.

1. Multistem Phosphorelay system involving AHKs, AHPs and ARRs [↑](#footnote-ref-2)
